# Supplementary figures and images for: Extracellular vesicle-mediated release of bis(monoacylglycerol)phosphate is regulated by LRRK2 and glucocerebrosidase activity
Source: eLife. 2026 Apr 2;14:RP106330. doi: 10.7554/eLife.106330 (PMC13046380; doi:10.7554/eLife.106330)

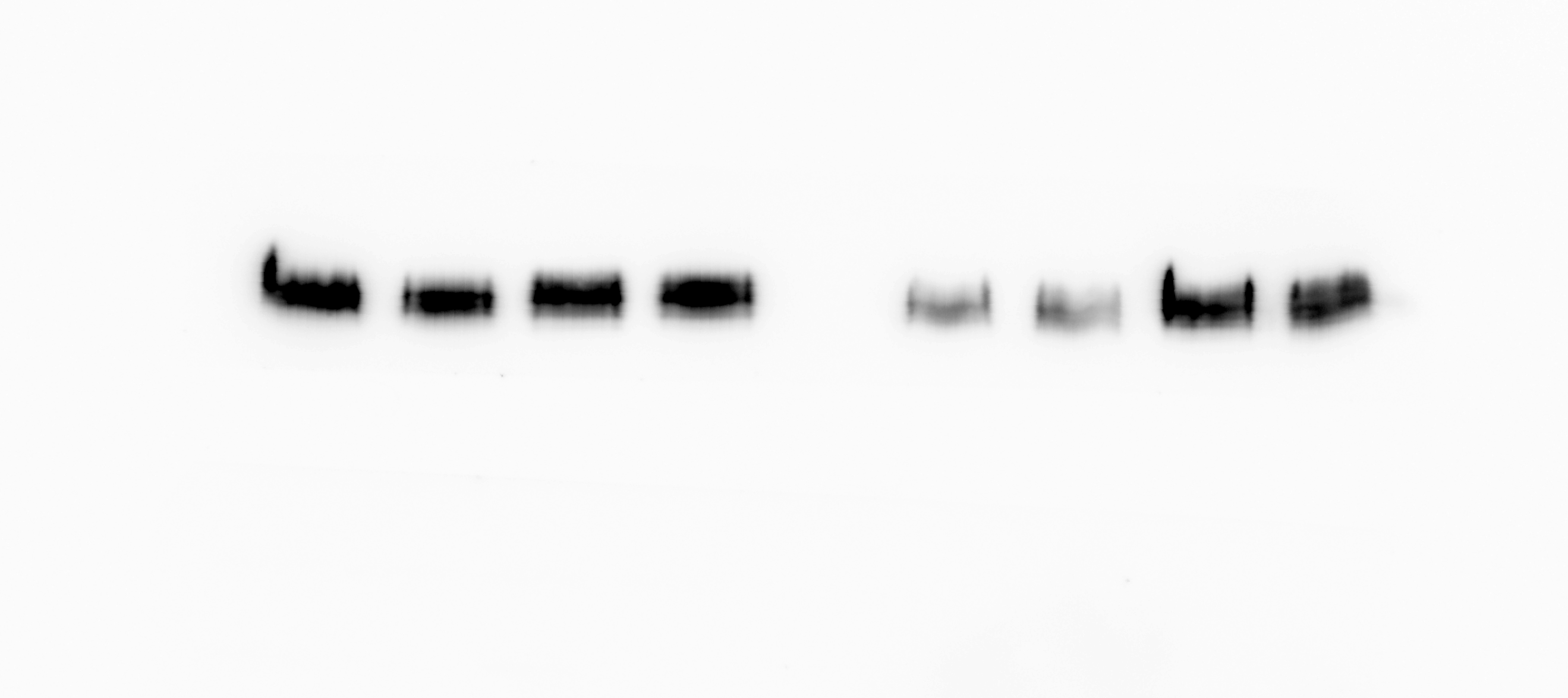

Supplement: Figure 2—source data 1. [file elife-106330-fig2-data1.zip › Figure 2A - Source data 1.tif]

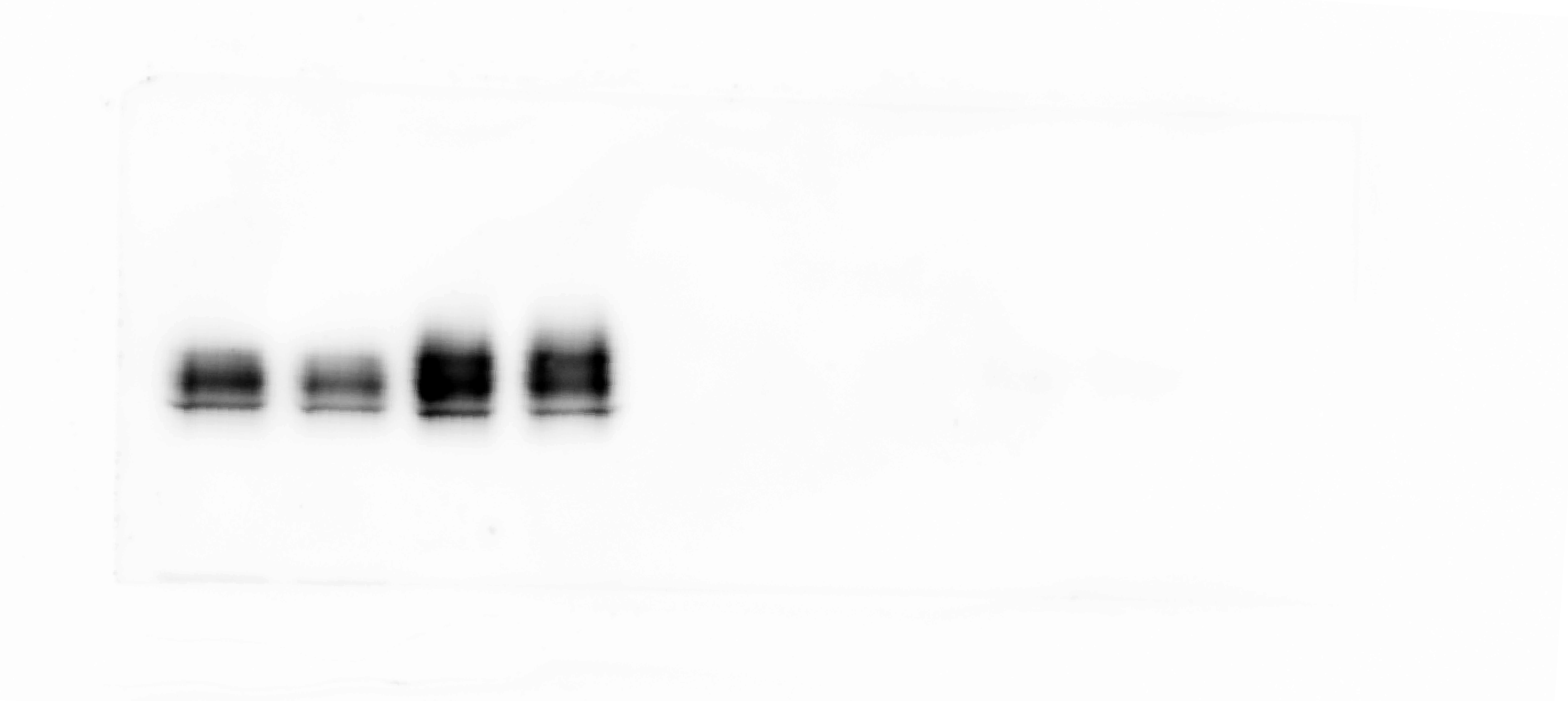

Supplement: Figure 2—source data 1. [file elife-106330-fig2-data1.zip › Figure 2C - Source data 1.tif]

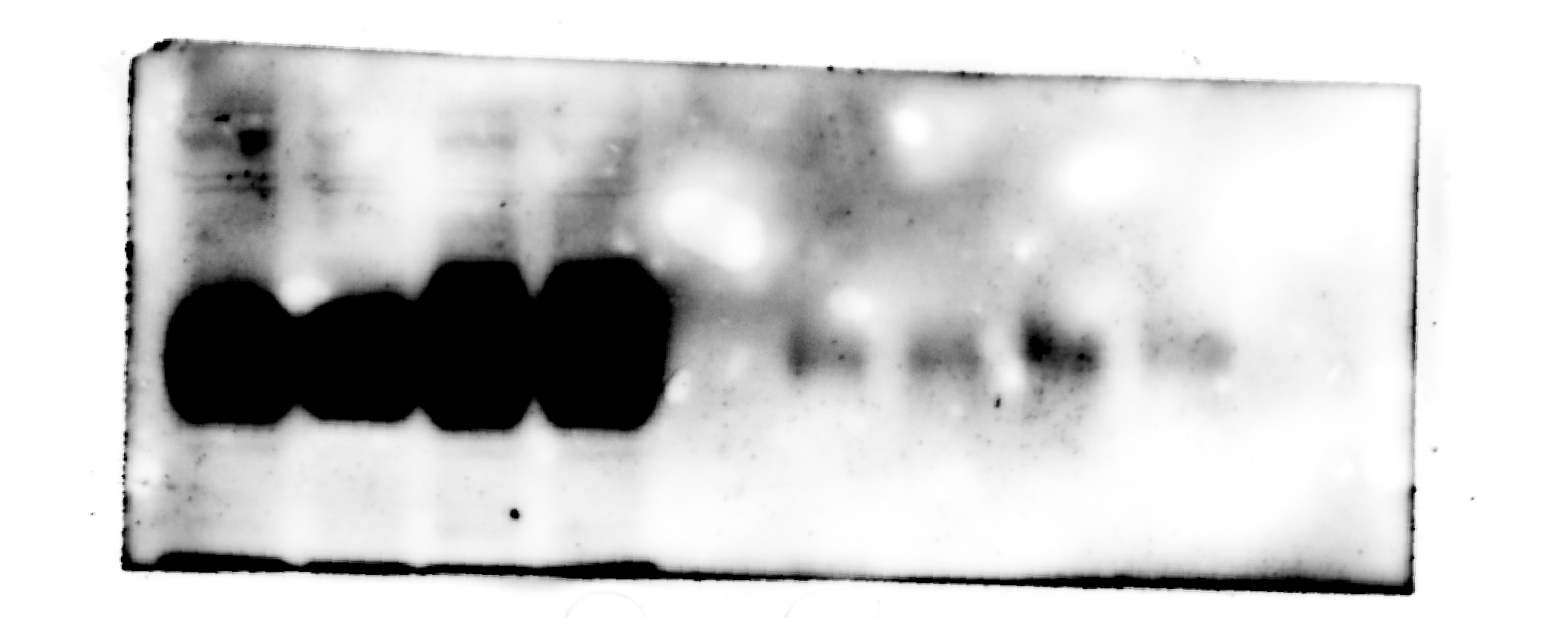

Supplement: Figure 2—source data 1. [file elife-106330-fig2-data1.zip › Figure 2C - Source data 2.tif]

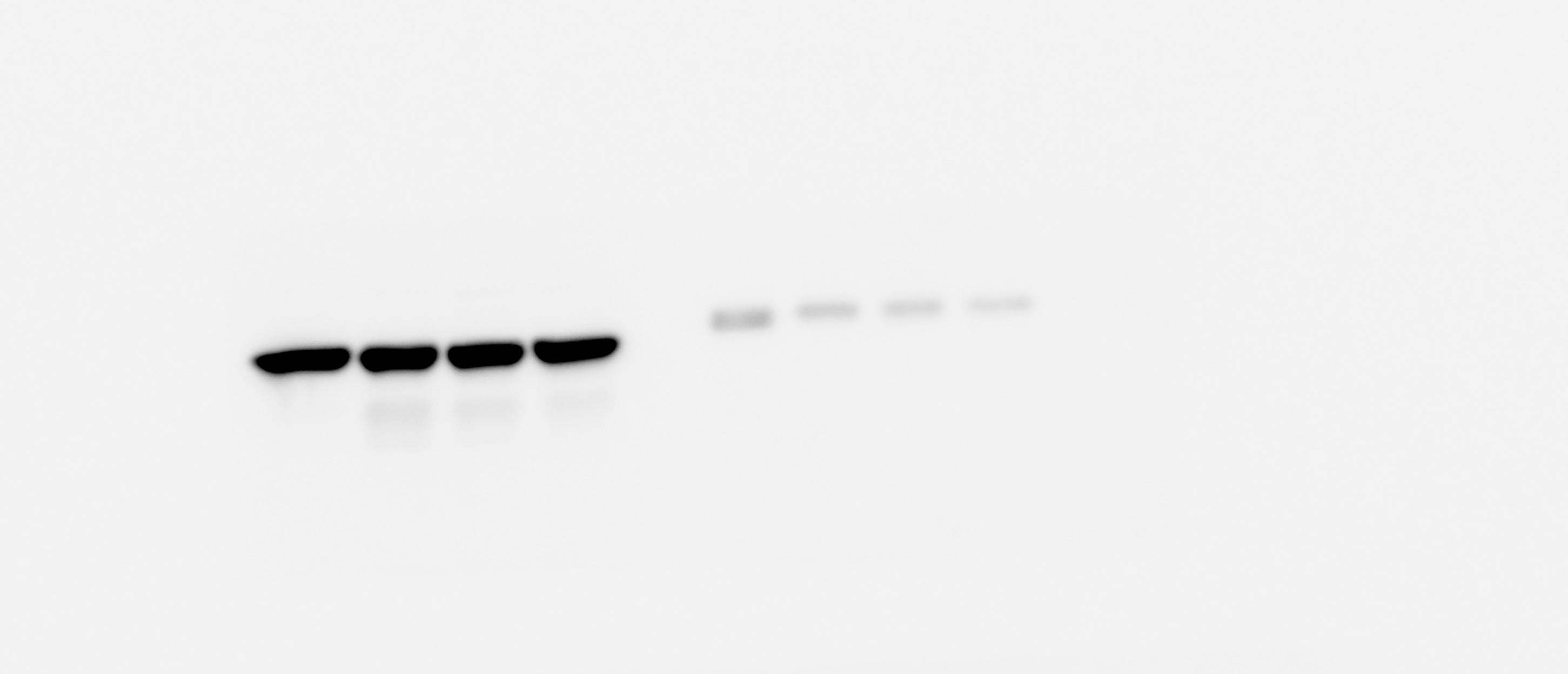

Supplement: Figure 2—source data 1. [file elife-106330-fig2-data1.zip › Figure 2C - Source data 3.tif]

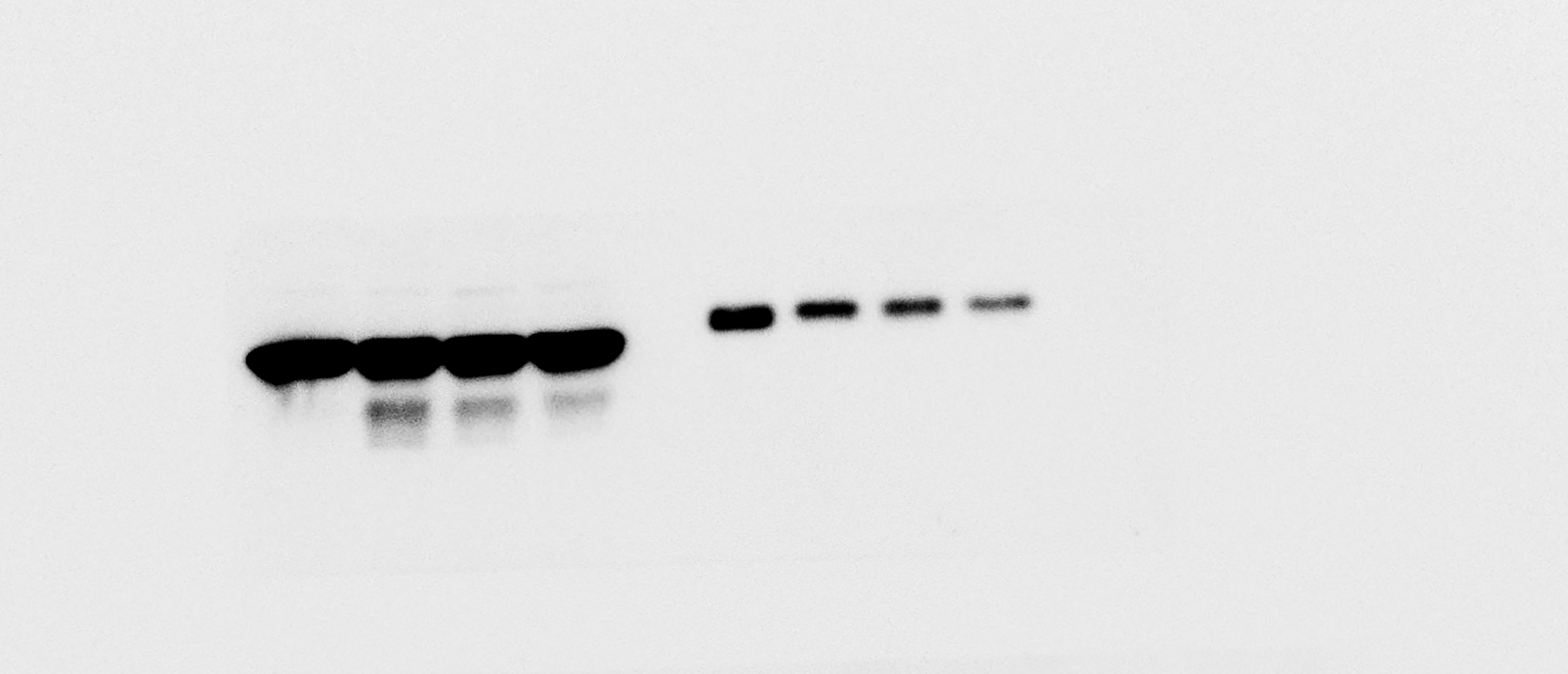

Supplement: Figure 2—source data 1. [file elife-106330-fig2-data1.zip › Figure 2C - Source data 4.tif]

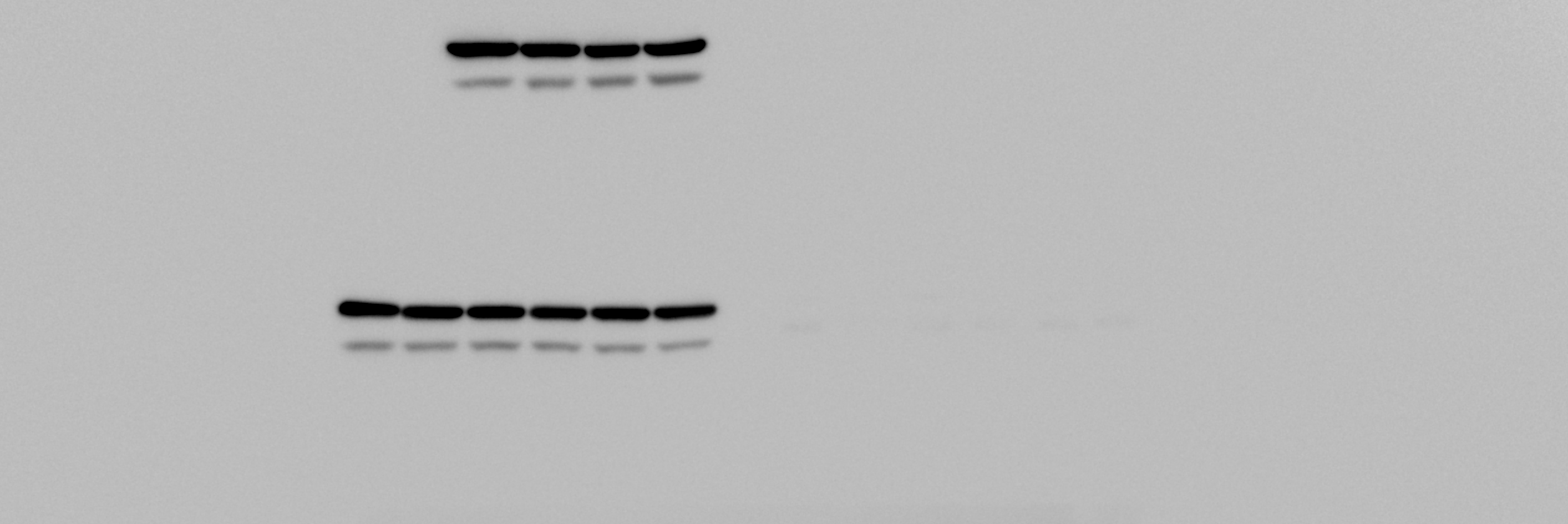

Supplement: Figure 2—source data 1. [file elife-106330-fig2-data1.zip › Figure 2C - Source data 5.tif]

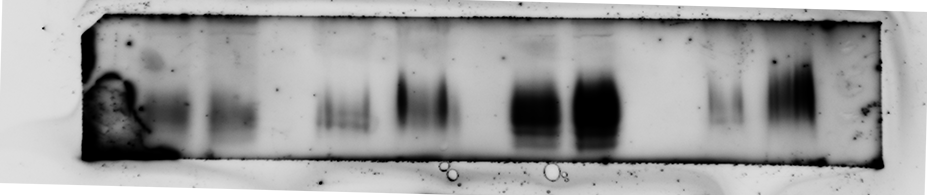

Supplement: Figure 2—source data 1. [file elife-106330-fig2-data1.zip › Figure 2G - Source data 1.tif]

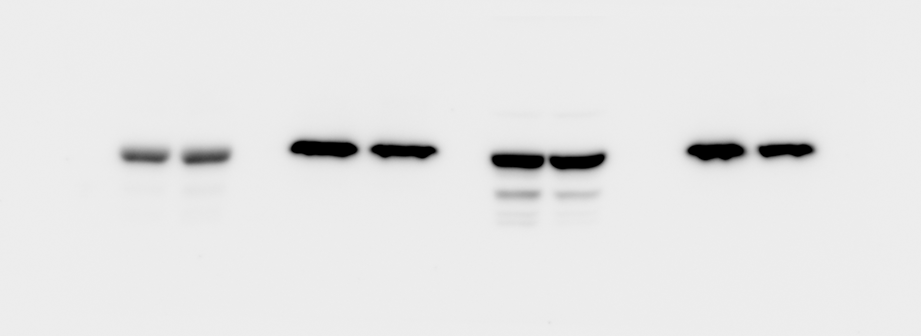

Supplement: Figure 2—source data 1. [file elife-106330-fig2-data1.zip › Figure 2G - Source data 2.tif]

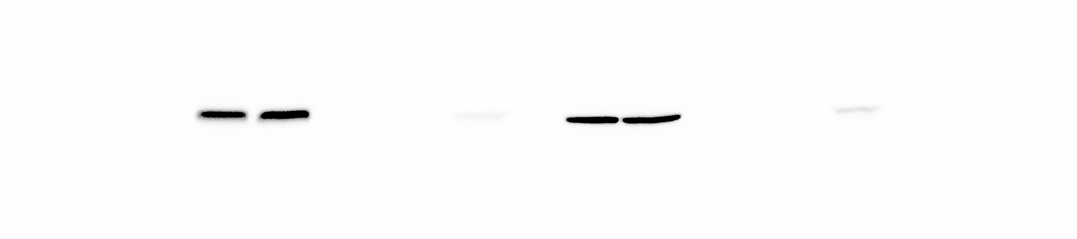

Supplement: Figure 2—source data 1. [file elife-106330-fig2-data1.zip › Figure 2G - Source data 3.tif]

Uncropped immunoblots for Figure 2 (Meneses-Salas et al.)

Figure 2A

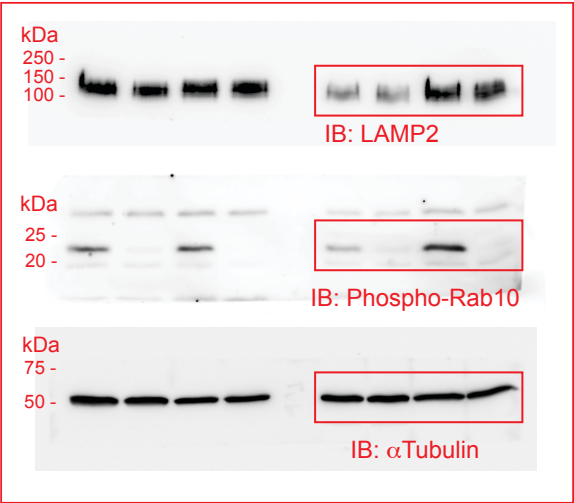

Figure 2C

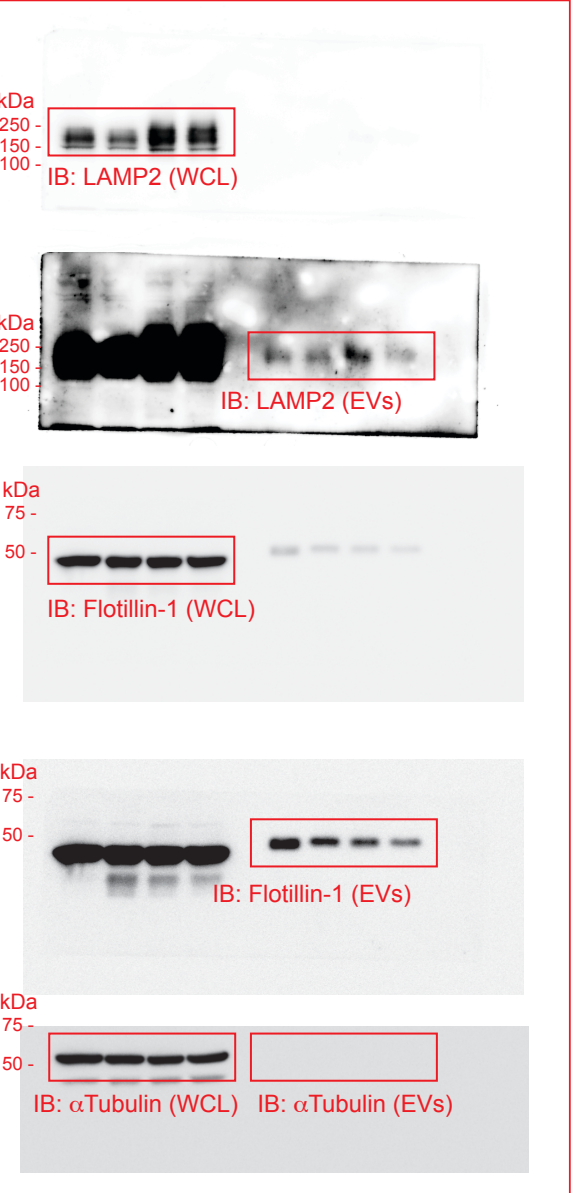

Figure 2G

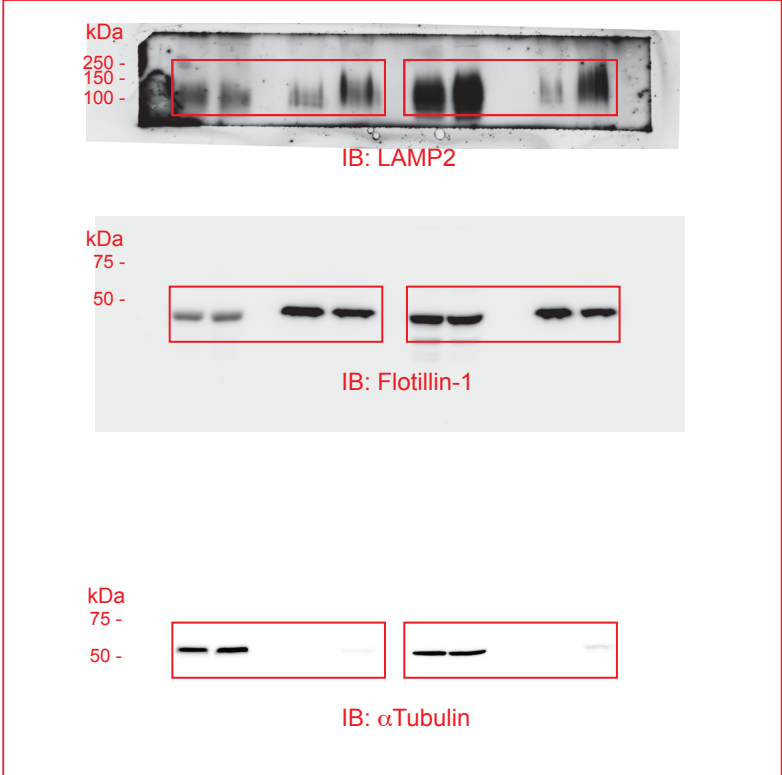

Supplement: Figure 2—source data 2. [file elife-106330-fig2-data2.zip › Figure 2 - Annotated Uncropped Blots.pdf]

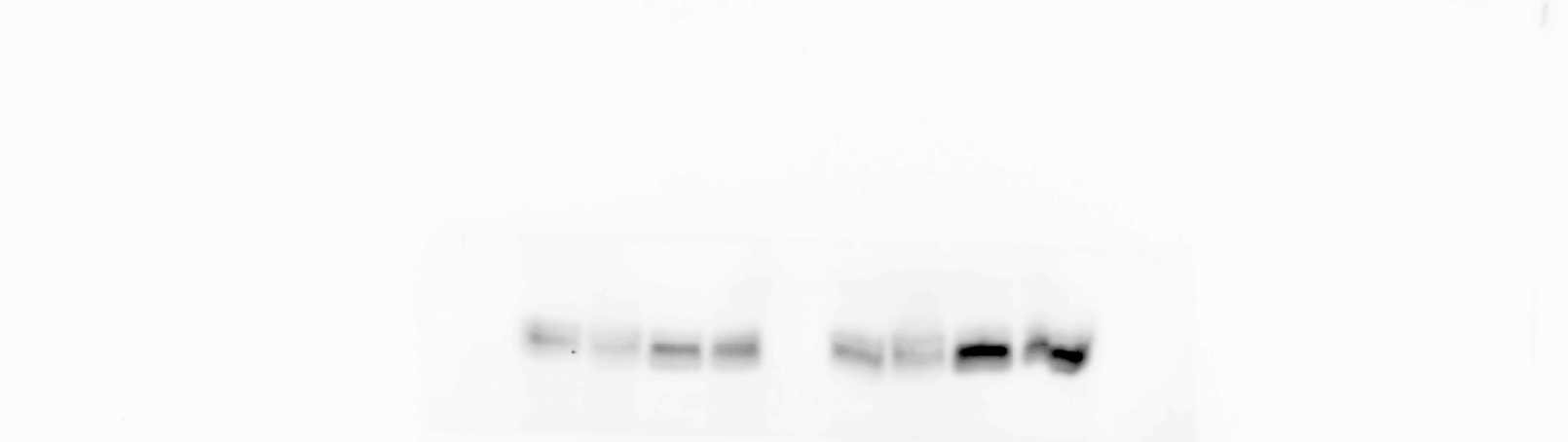

Supplement: Figure 5—source data 1. [file elife-106330-fig5-data1.zip › Figure 5A - Source data 1.tif]

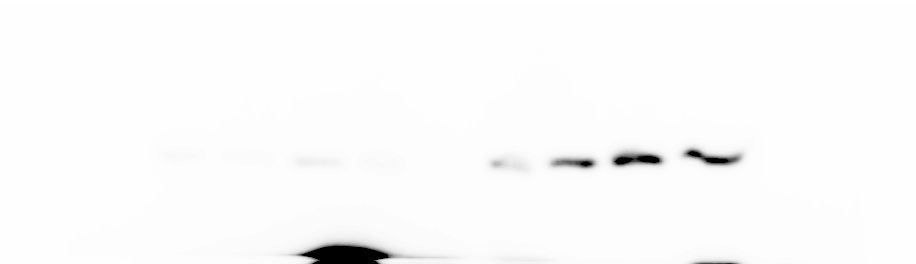

Supplement: Figure 5—source data 1. [file elife-106330-fig5-data1.zip › Figure 5A - Source data 2.tif]

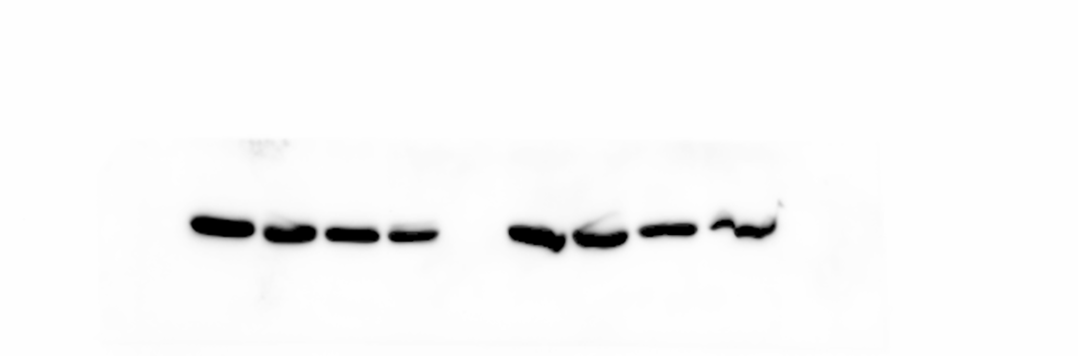

Supplement: Figure 5—source data 1. [file elife-106330-fig5-data1.zip › Figure 5A - Source data 3.tif]

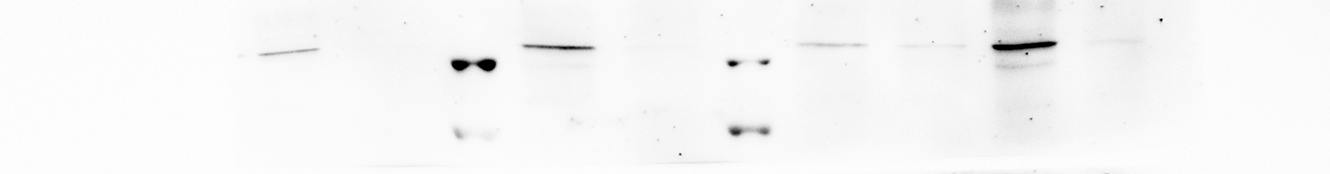

Supplement: Figure 5—source data 1. [file elife-106330-fig5-data1.zip › Figure 5B - Source data 1.tif]

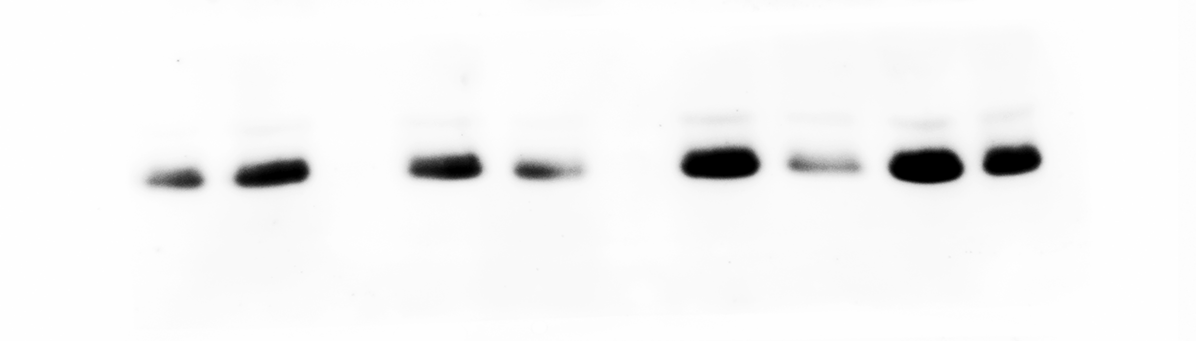

Supplement: Figure 5—source data 1. [file elife-106330-fig5-data1.zip › Figure 5B - Source data 2.tif]

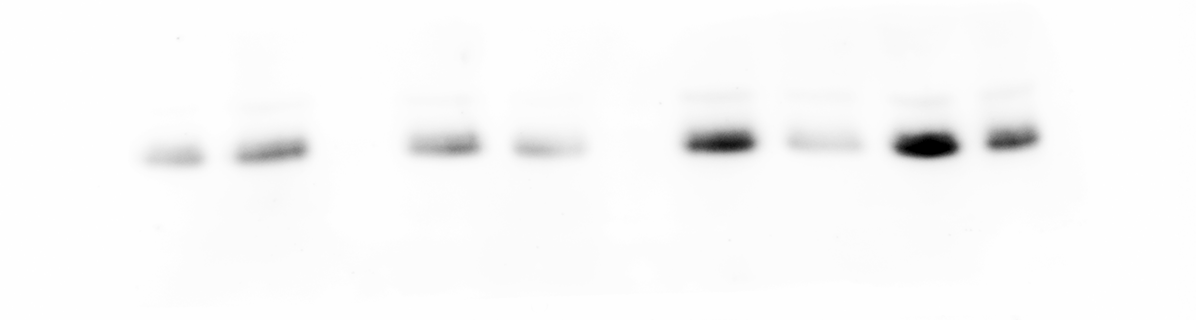

Supplement: Figure 5—source data 1. [file elife-106330-fig5-data1.zip › Figure 5B - Source data 3.tif]

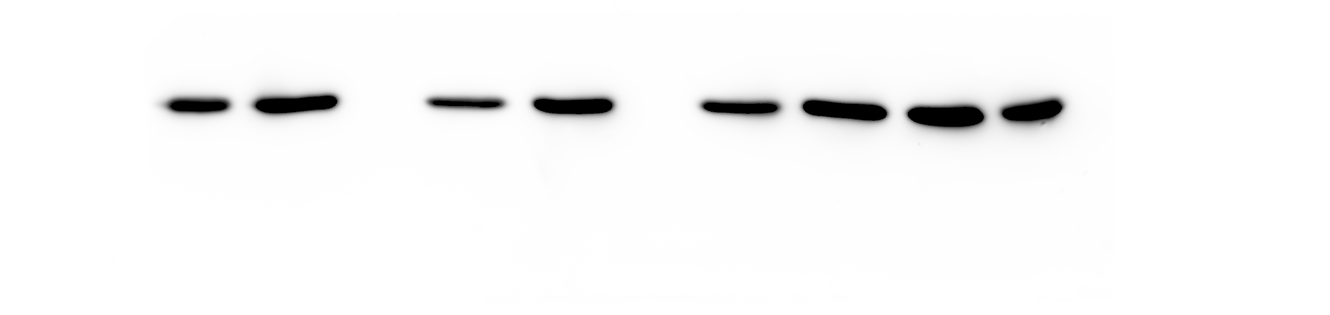

Supplement: Figure 5—source data 1. [file elife-106330-fig5-data1.zip › Figure 5B - Source data 4.tif]

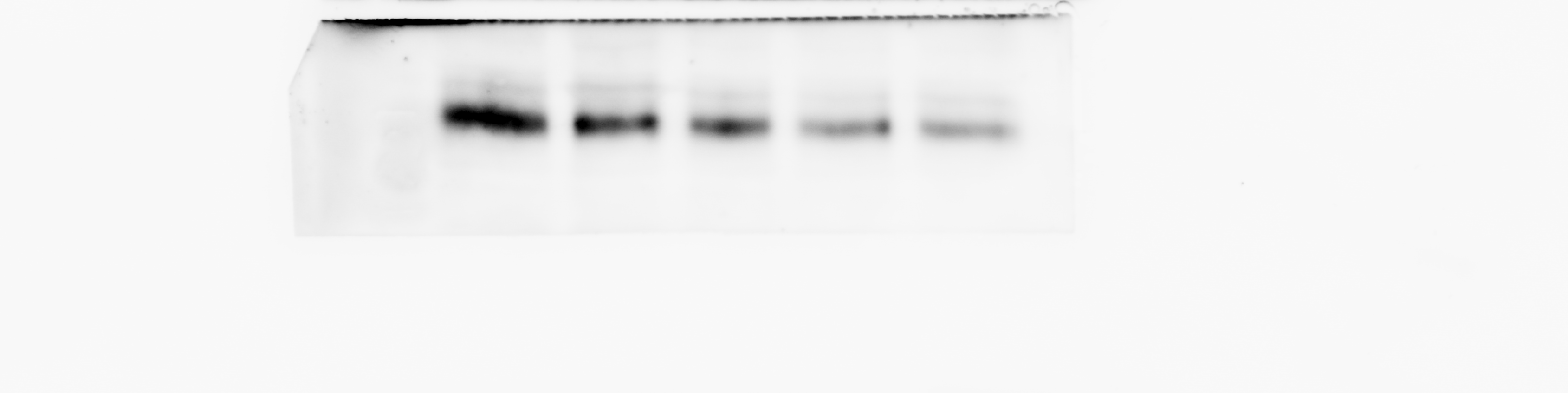

Supplement: Figure 5—source data 1. [file elife-106330-fig5-data1.zip › Figure 5C - Source data 1.tif]

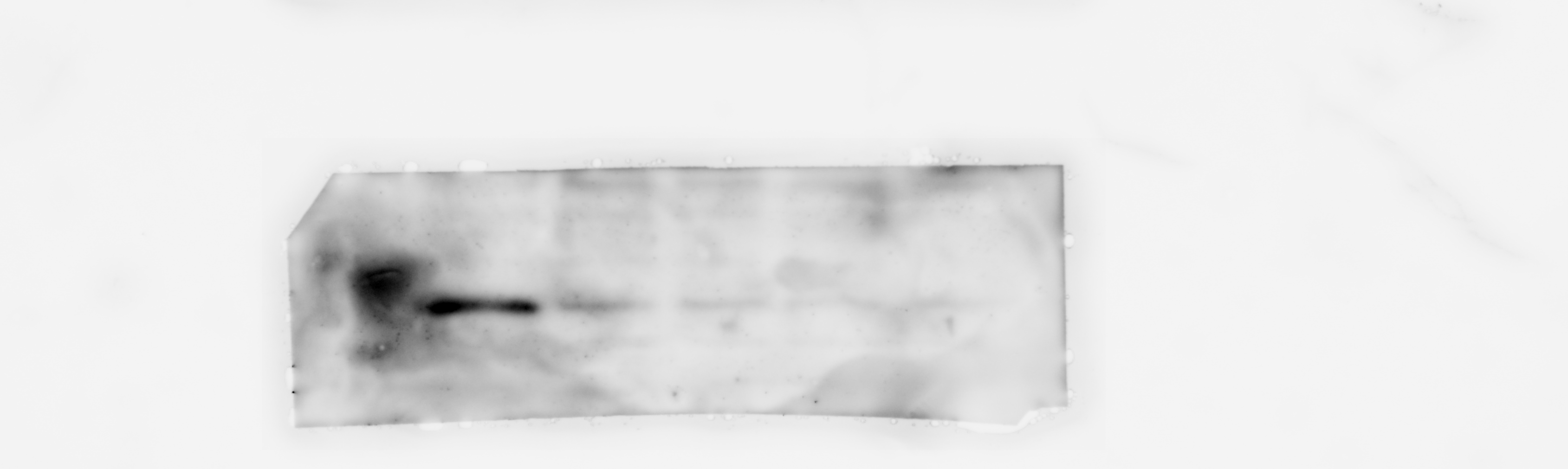

Supplement: Figure 5—source data 1. [file elife-106330-fig5-data1.zip › Figure 5C - Source data 2.tif]

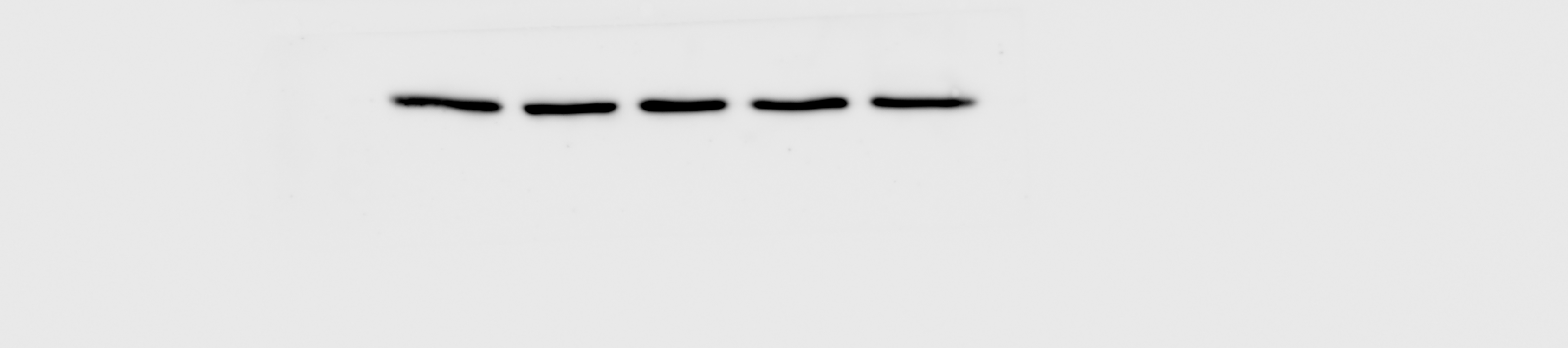

Supplement: Figure 5—source data 1. [file elife-106330-fig5-data1.zip › Figure 5C - Source data 3.tif]

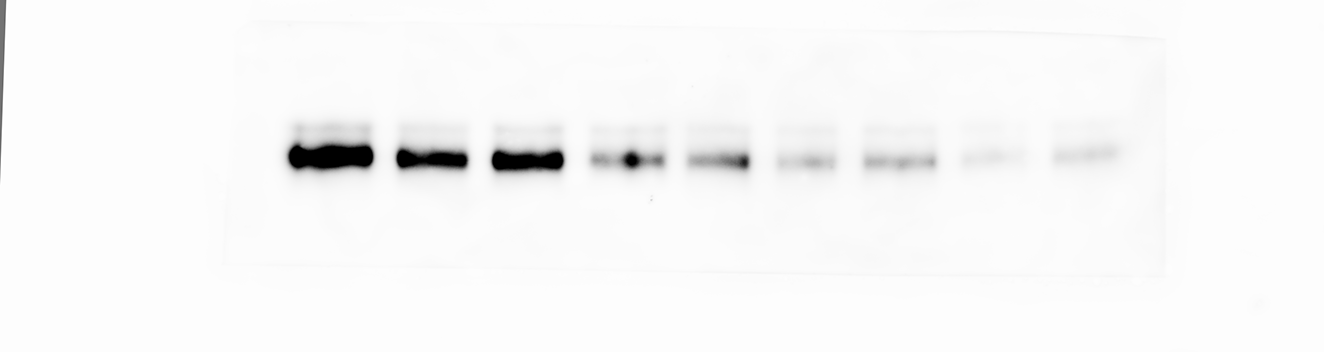

Supplement: Figure 5—source data 1. [file elife-106330-fig5-data1.zip › Figure 5D - Source data 1.tif]

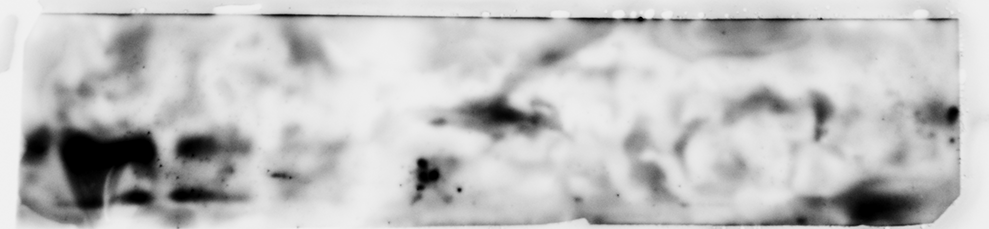

Supplement: Figure 5—source data 1. [file elife-106330-fig5-data1.zip › Figure 5D - Source data 2.tif]

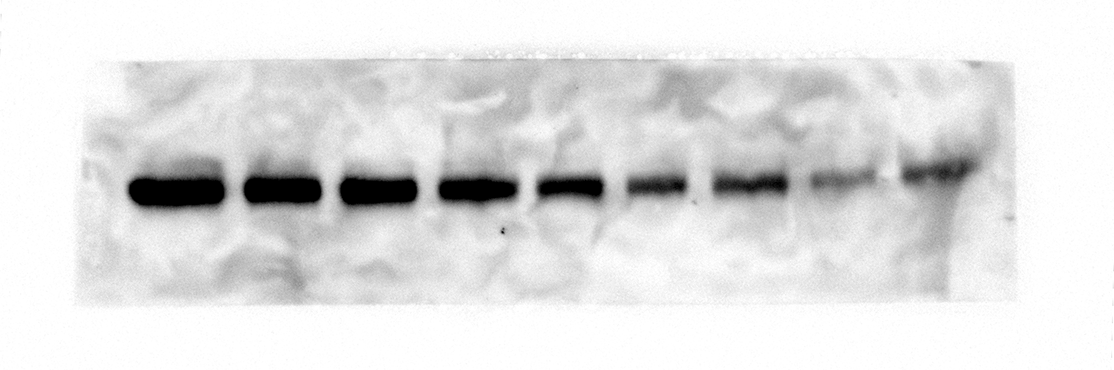

Supplement: Figure 5—source data 1. [file elife-106330-fig5-data1.zip › Figure 5D - Source data 3.tif]

Uncropped immunoblots for Figure 5 (Meneses-Salas et al.)

Figure 5A

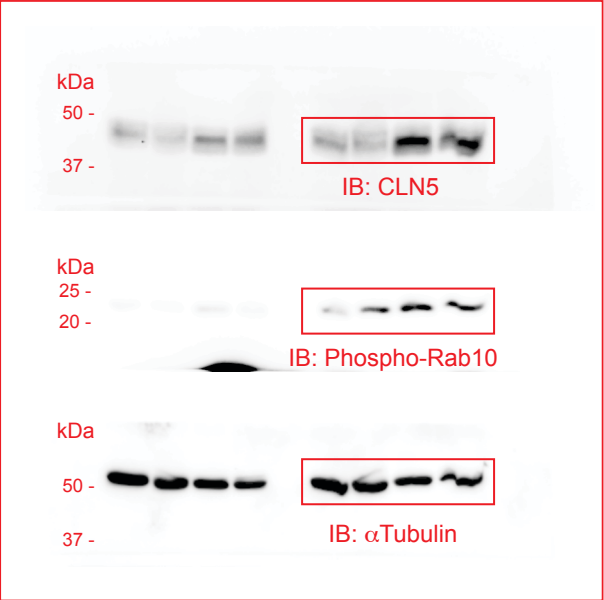

Figure 5B

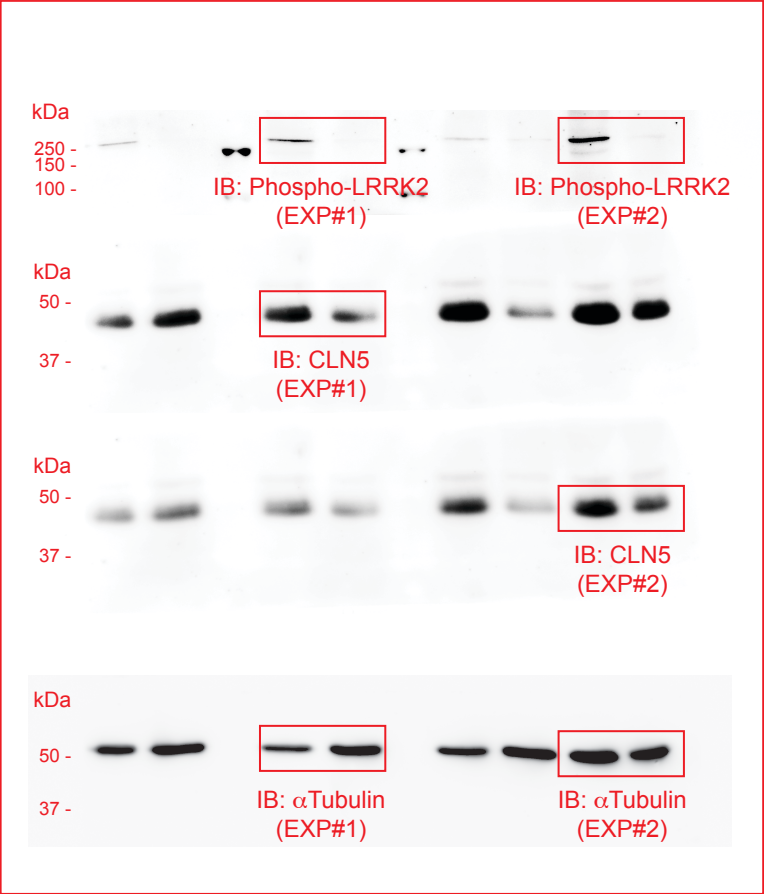

Figure 5C

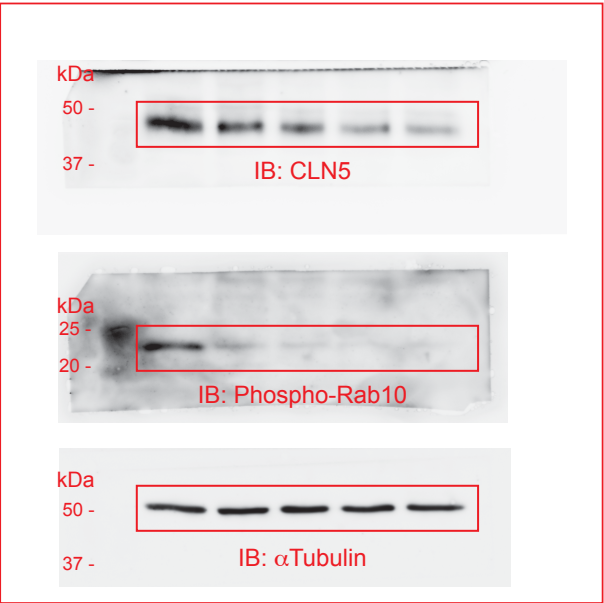

Figure 5D

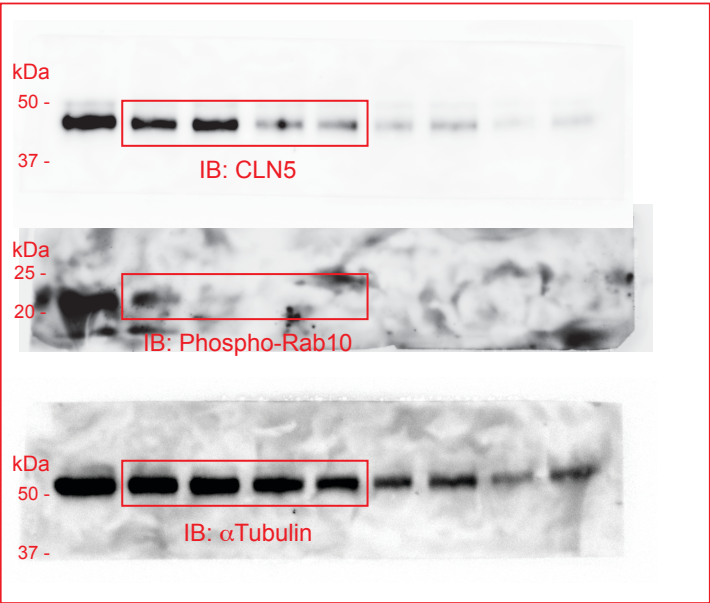

Supplement: Figure 5—source data 2. [file elife-106330-fig5-data2.zip › Figure 5 - Annotated Uncropped Blots.pdf]

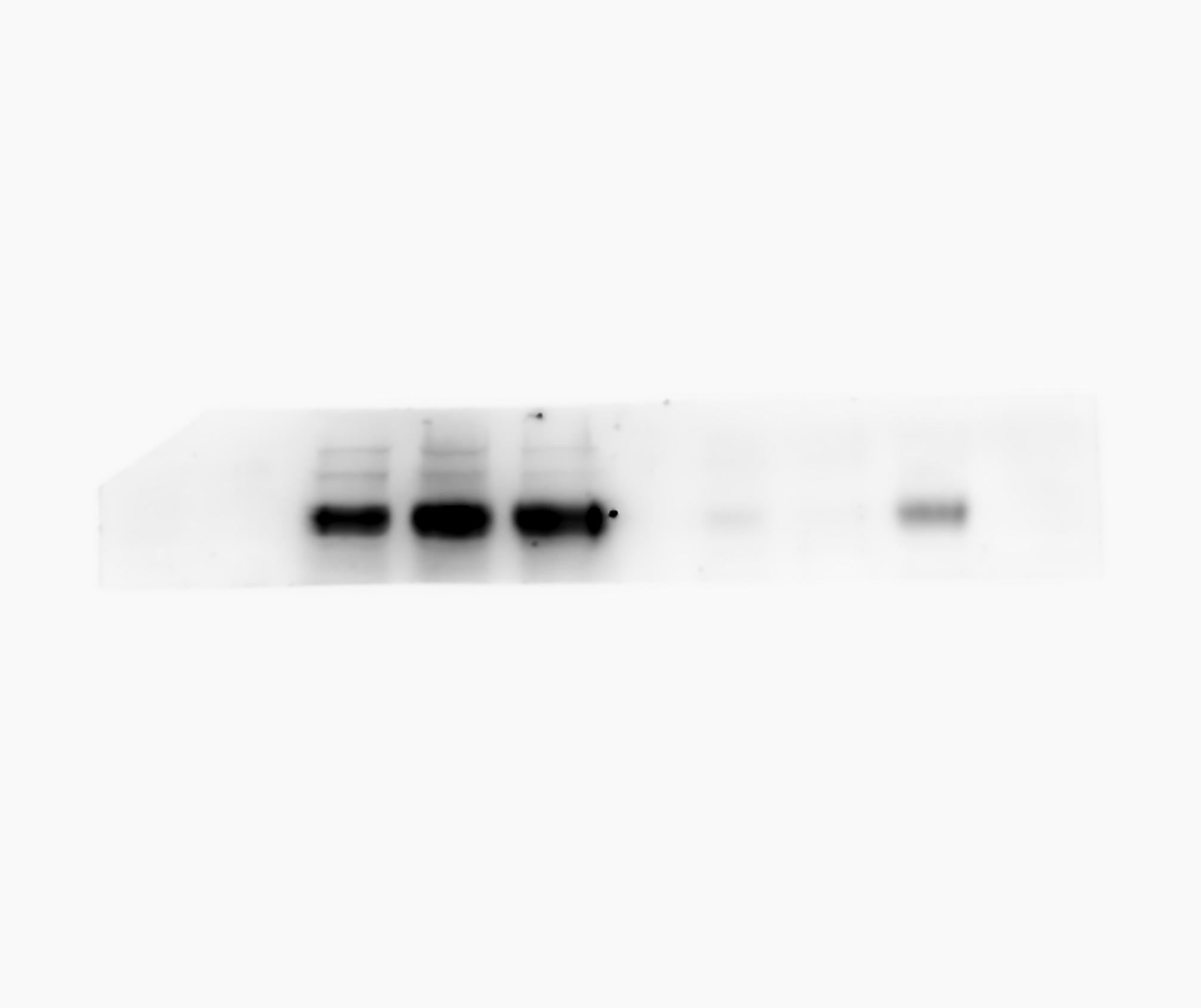

Supplement: Figure 6—source data 1. [file elife-106330-fig6-data1.zip › Figure 6A - Source data 1.tif]

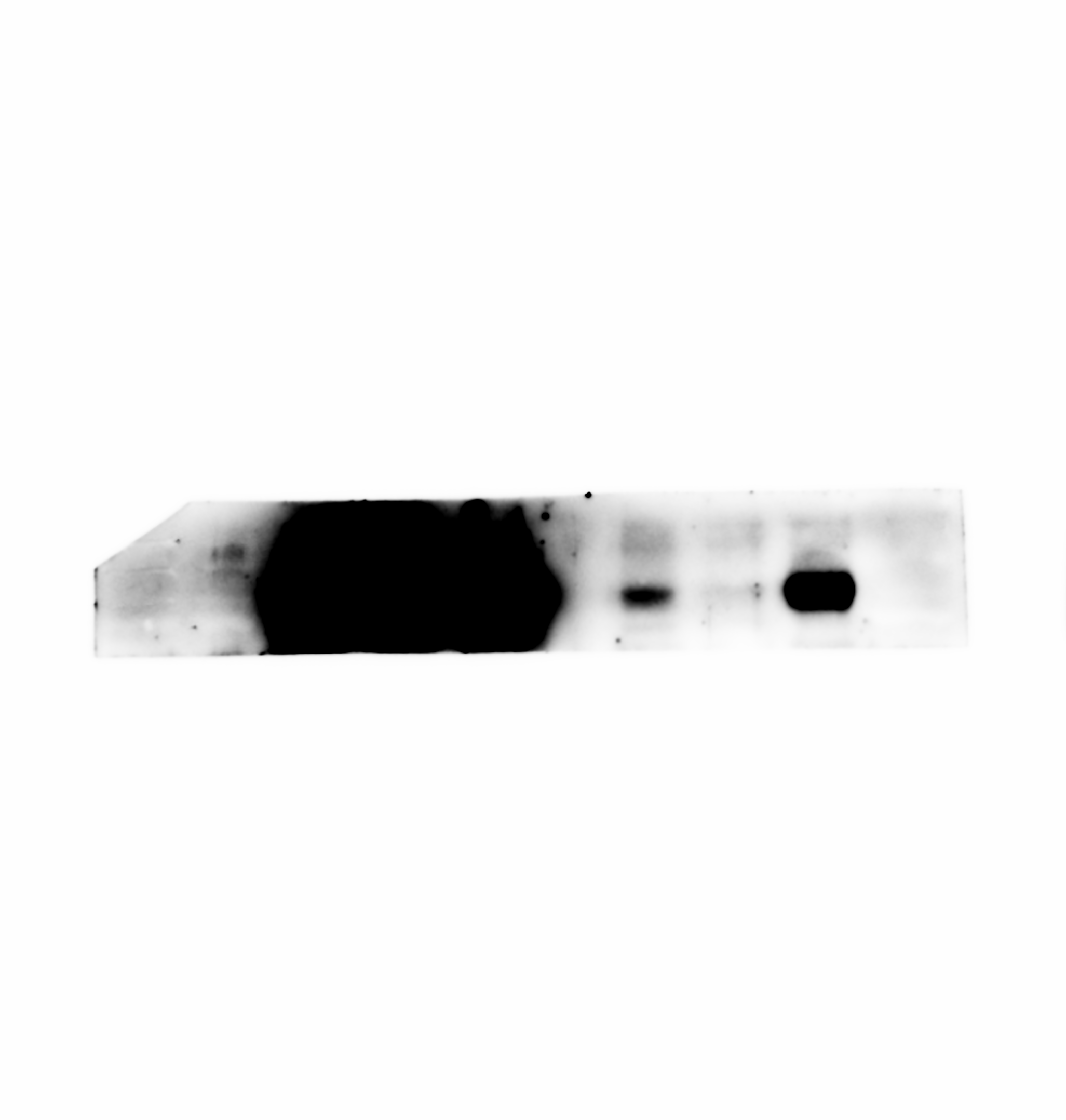

Supplement: Figure 6—source data 1. [file elife-106330-fig6-data1.zip › Figure 6A - Source data 2.tif]

Uncropped immunoblots for Figure 6 (Meneses-Salas et al.)

Figure 6A

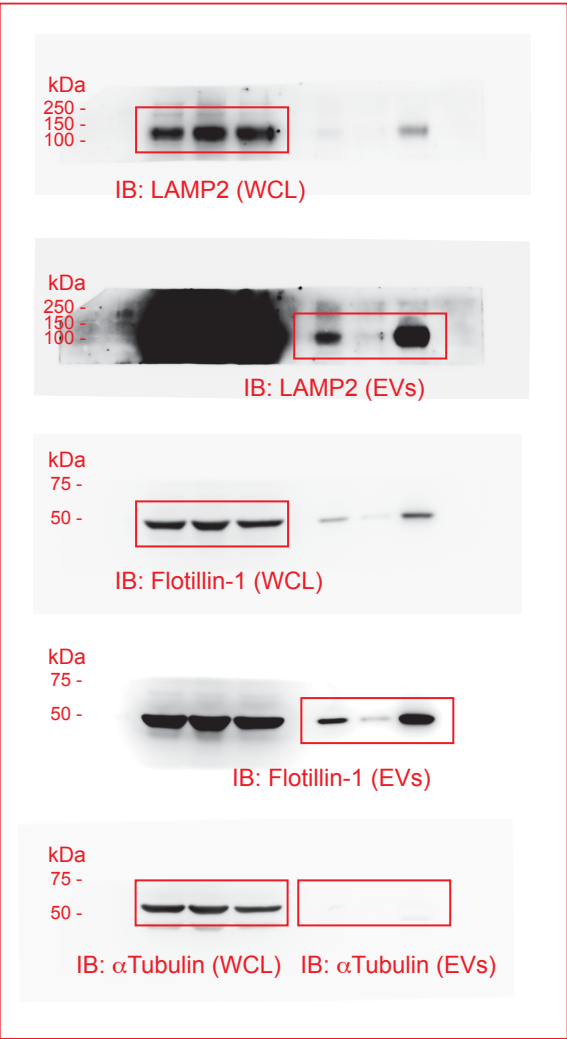

Supplement: Figure 6—source data 2. [file elife-106330-fig6-data2.zip › Figure 6 - Annotated Uncropped Blots.pdf]

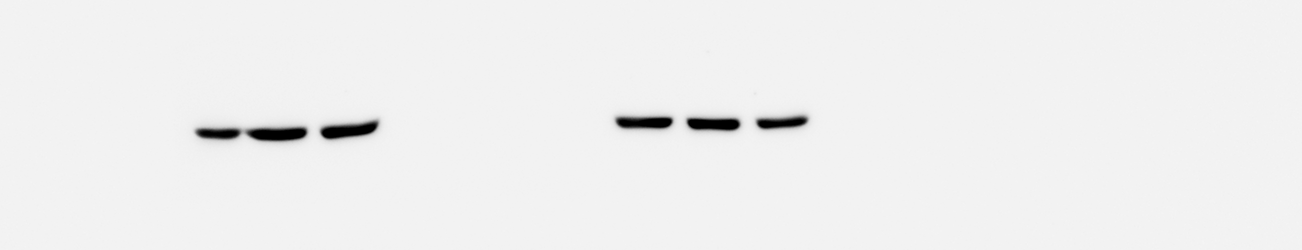

Supplement: Figure 6—figure supplement 1—source data 1. [file elife-106330-fig6-figsupp1-data1.zip › Figure 6-figure supplement 1 - Source data 5 WB.tif]

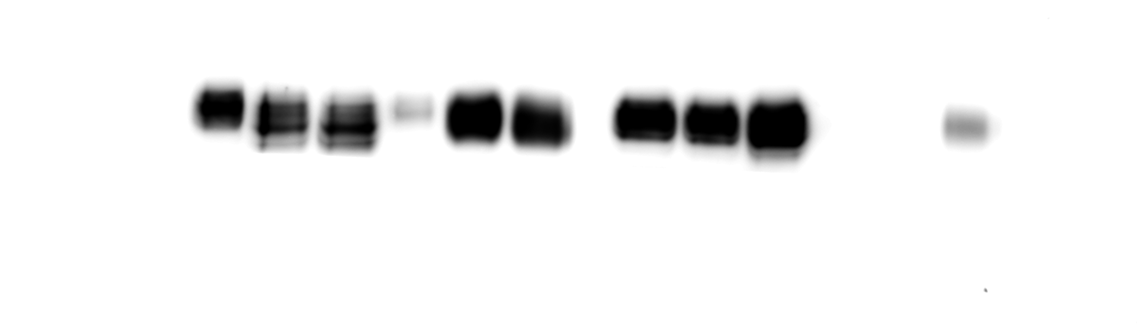

Supplement: Figure 6—figure supplement 1—source data 1. [file elife-106330-fig6-figsupp1-data1.zip › Figure 6-figure supplement 1 - Source data 1 WB.tif]

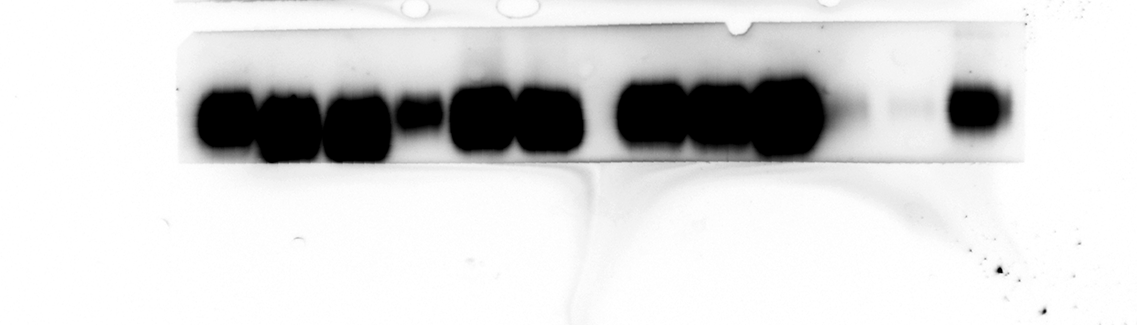

Supplement: Figure 6—figure supplement 1—source data 1. [file elife-106330-fig6-figsupp1-data1.zip › Figure 6-figure supplement 1 - Source data 2 WB.tif]

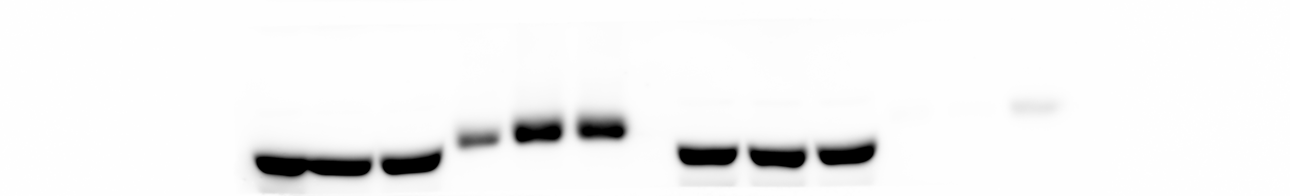

Supplement: Figure 6—figure supplement 1—source data 1. [file elife-106330-fig6-figsupp1-data1.zip › Figure 6-figure supplement 1 - Source data 3 WB.tif]

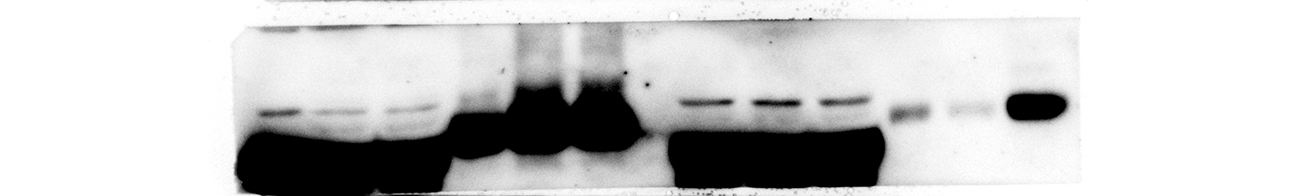

Supplement: Figure 6—figure supplement 1—source data 1. [file elife-106330-fig6-figsupp1-data1.zip › Figure 6-figure supplement 1 - Source data 4 WB.tif]

Uncropped immunoblots for Figure 6-figure supplement 1 (Meneses-Salas et al.)

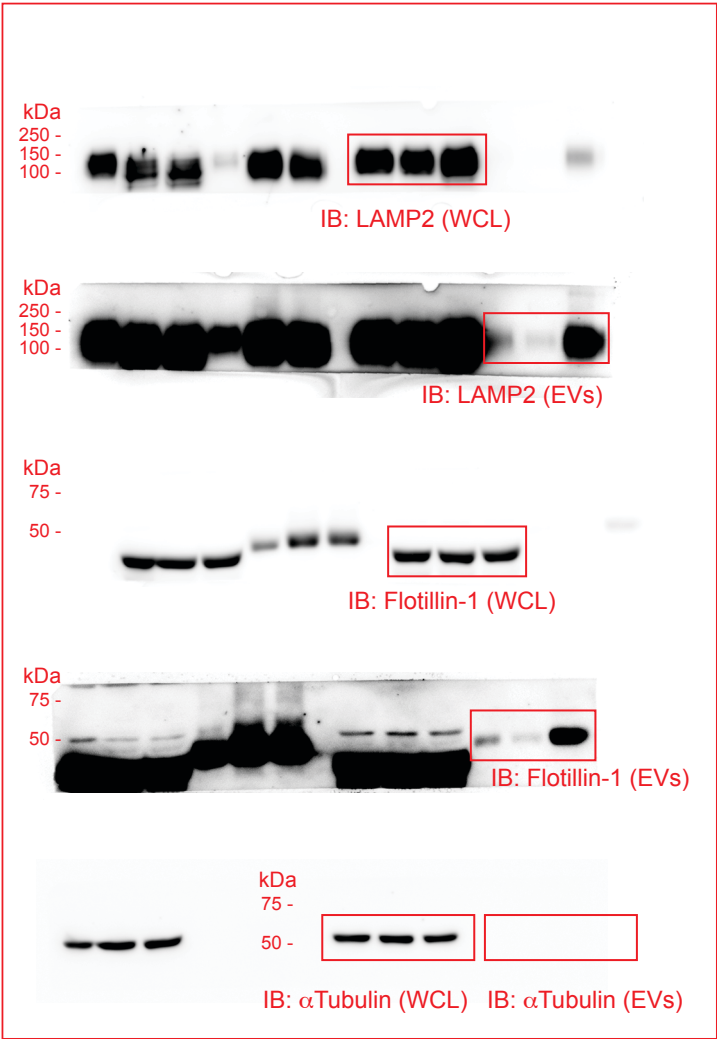

Supplement: Figure 6—figure supplement 1—source data 2. [file elife-106330-fig6-figsupp1-data2.zip › Figure 6-figure supplement 1 - Annotated Uncropped Blots.pdf]

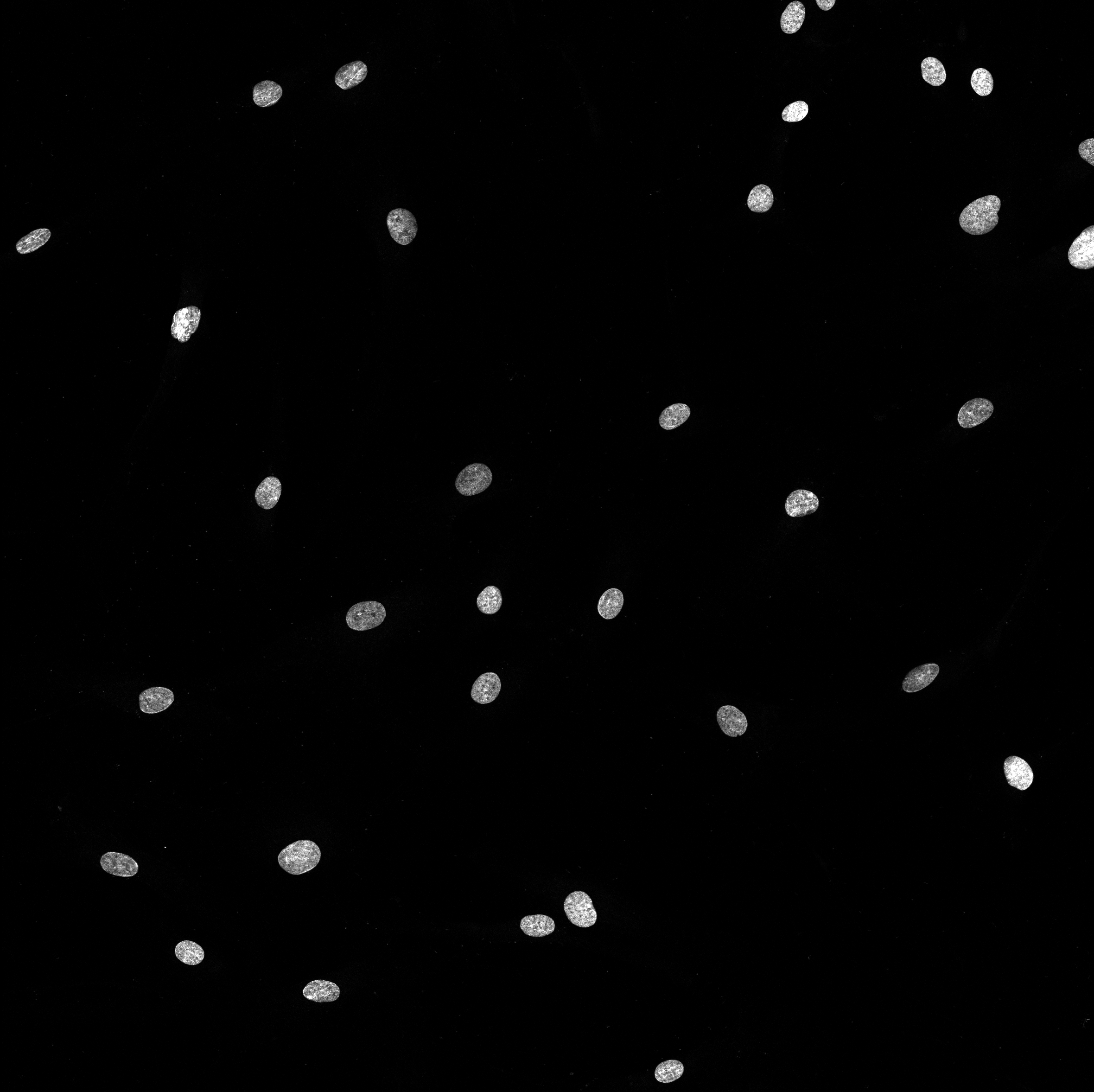

Supplement: Figure 7—source data 1. [file elife-106330-fig7-data1.zip › Figure 7A - Source data 1_IF CTRL.tif]

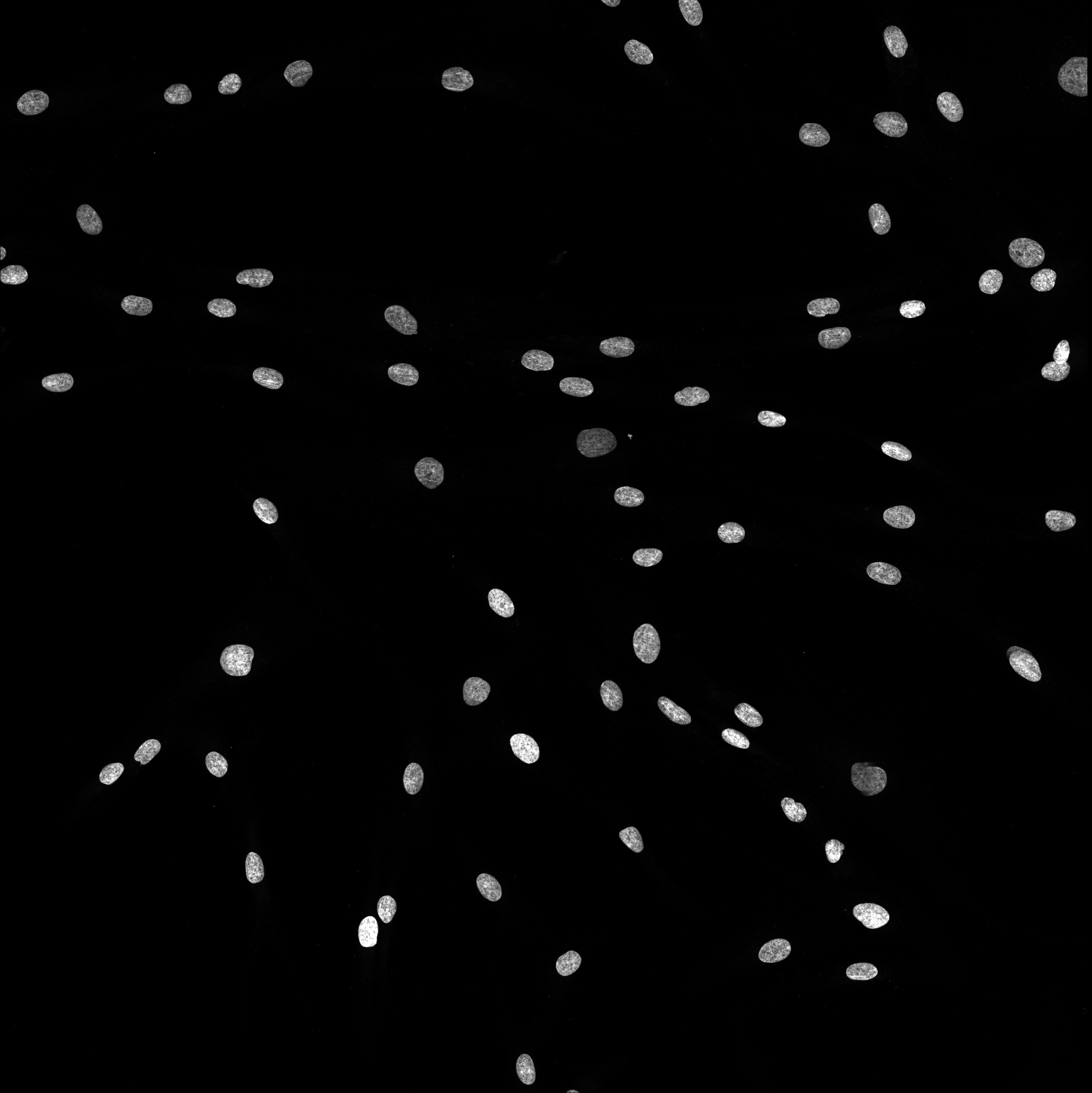

Supplement: Figure 7—source data 1. [file elife-106330-fig7-data1.zip › Figure 7A - Source data 2_IF G2019S LRRK2.tif]

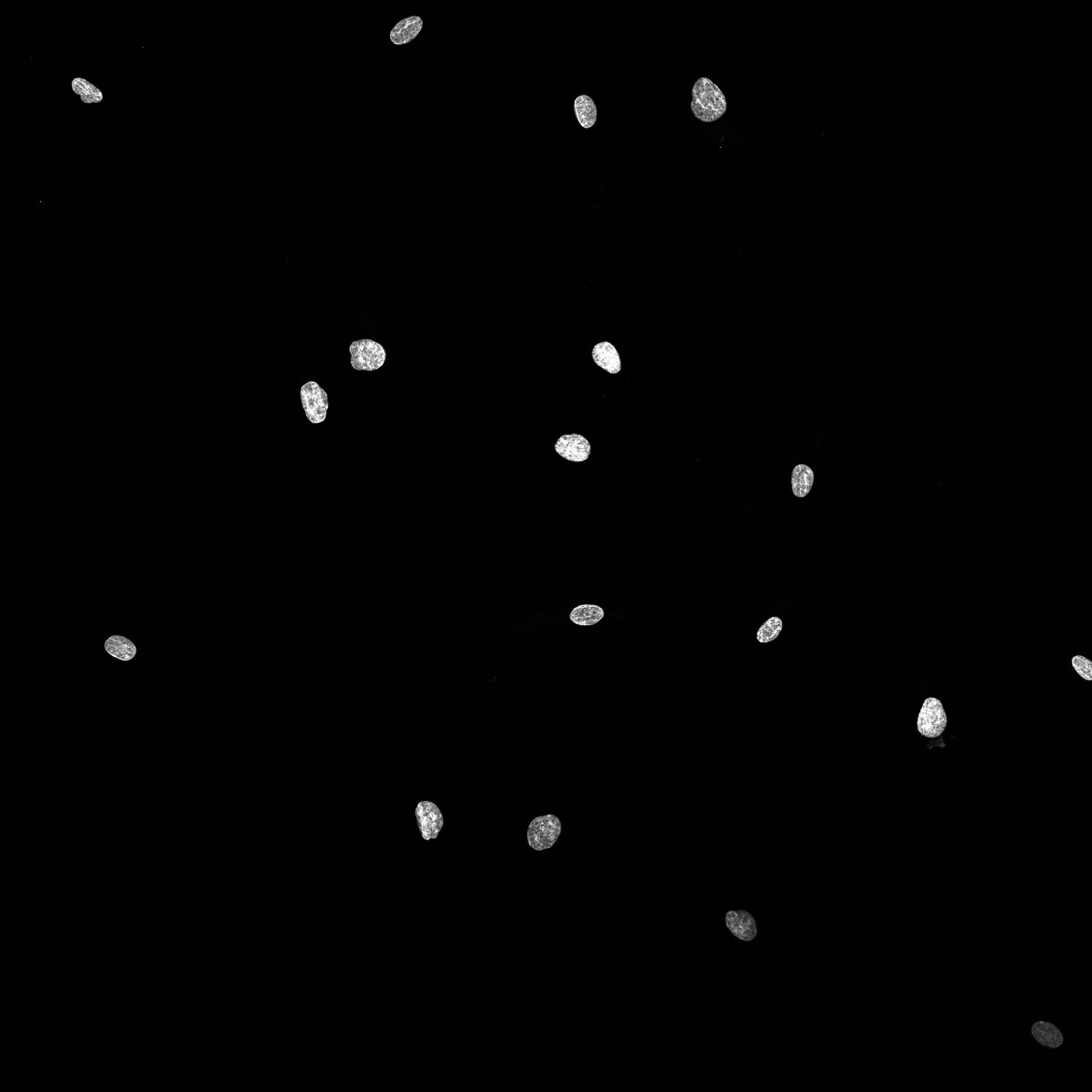

Supplement: Figure 7—source data 2. [file elife-106330-fig7-data2.zip › Figure 7D - Source data 1_IF CTRL.tif]

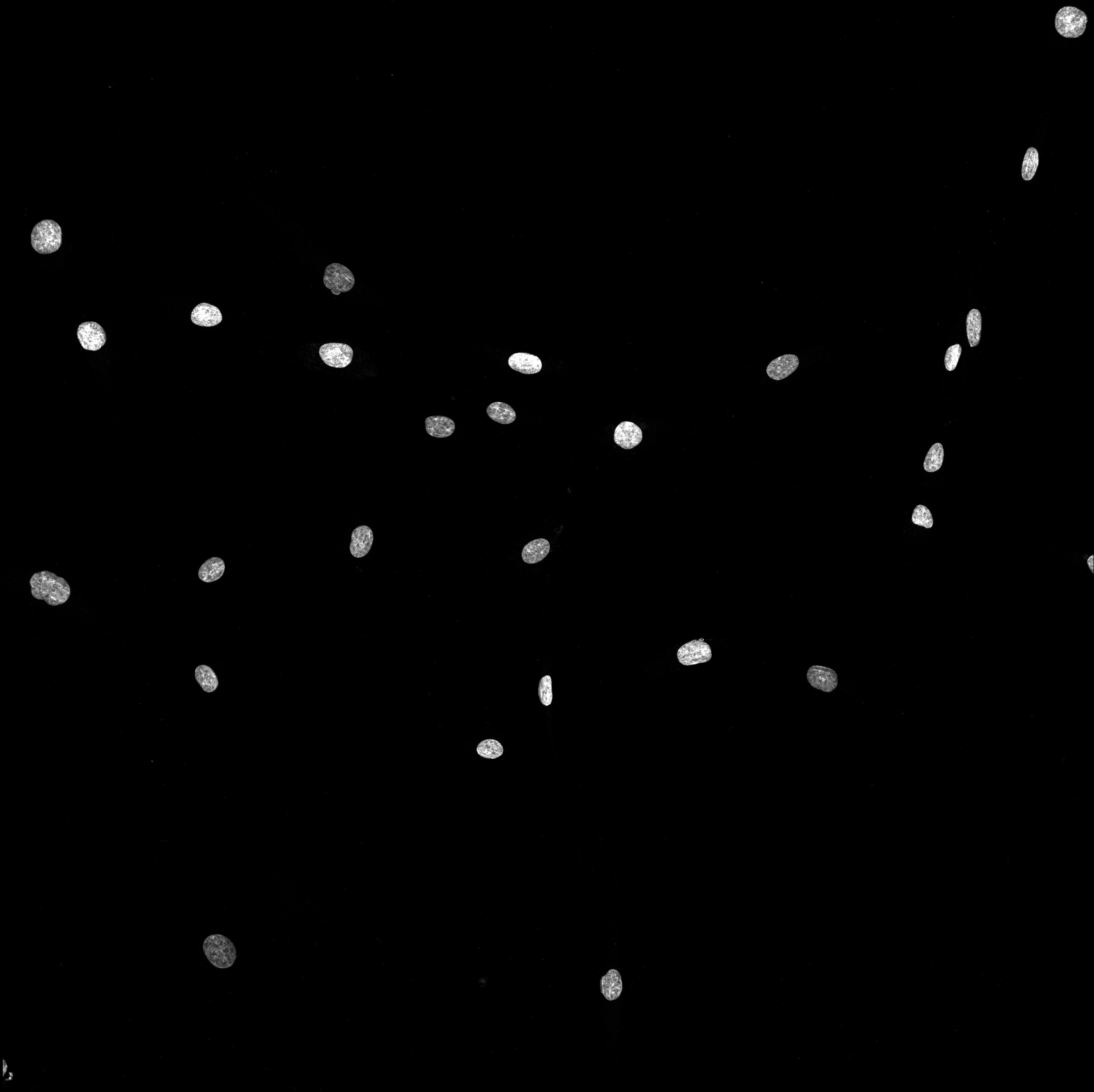

Supplement: Figure 7—source data 2. [file elife-106330-fig7-data2.zip › Figure 7D - Source data 2_IF G2019S LRRK2.tif]

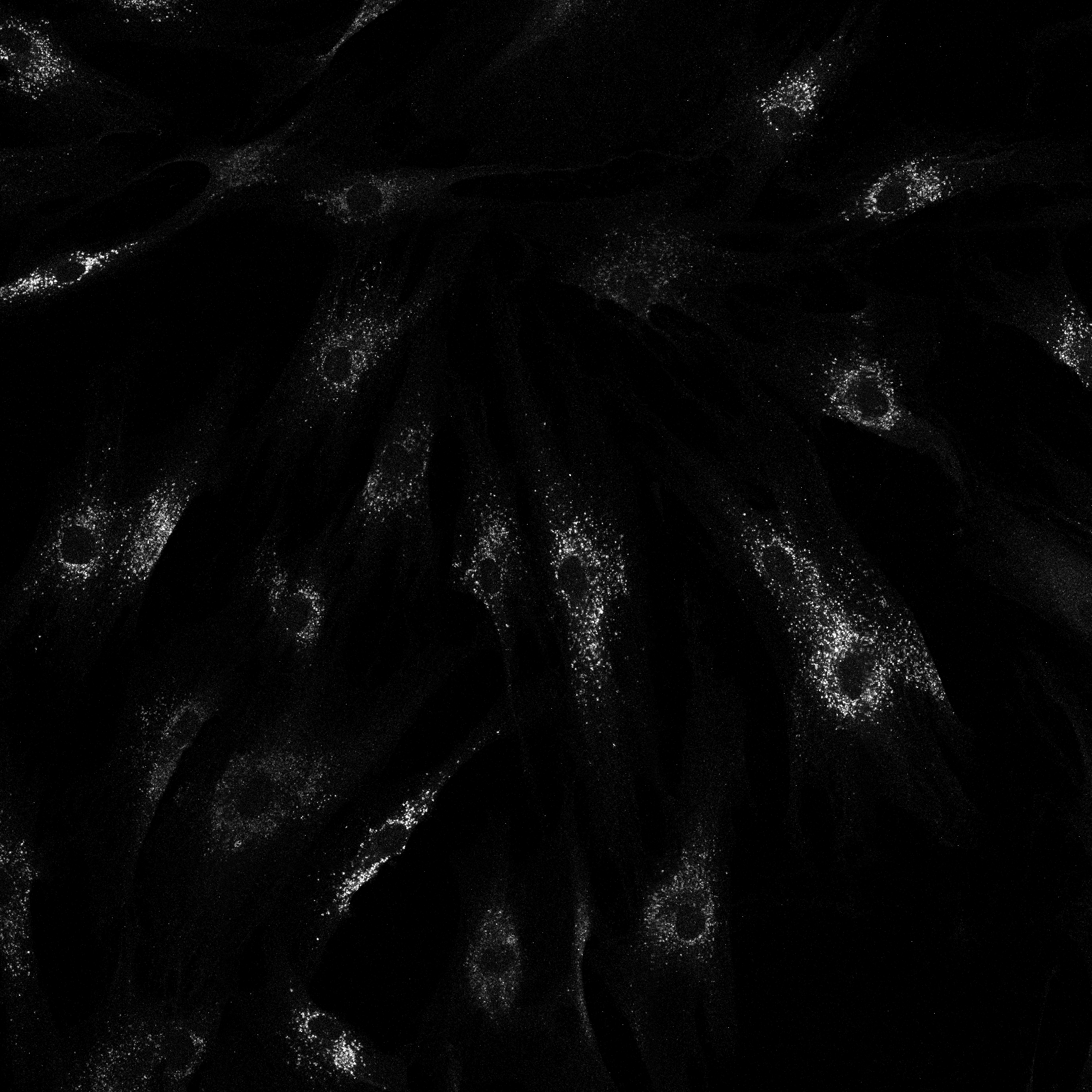

Supplement: Figure 7—source data 3. [file elife-106330-fig7-data3.zip › Figure 7G - Source data 1_IF.tif]

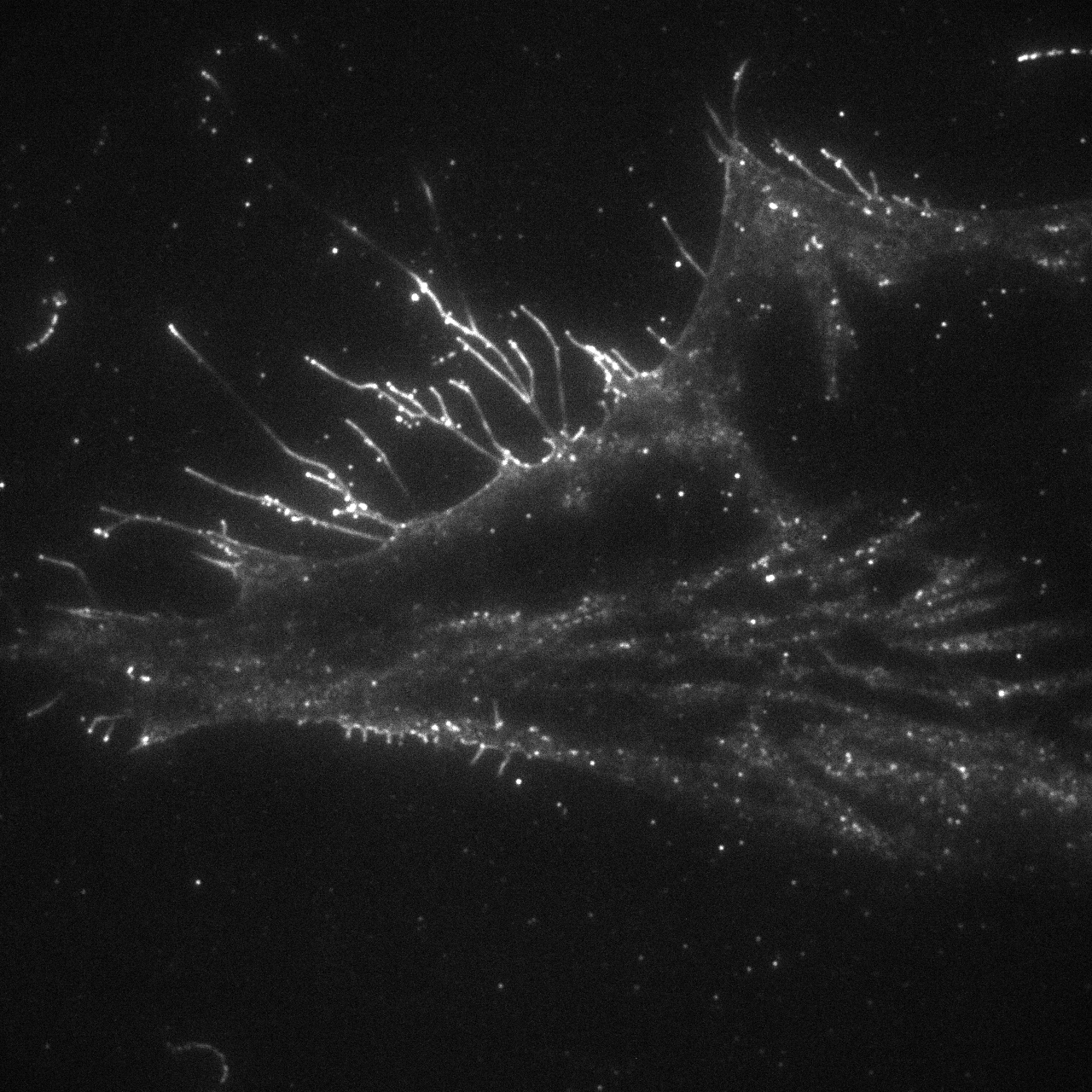

Supplement: Figure 7—source data 4. [file elife-106330-fig7-data4.zip › Figure 7H - Source data 2_TIRF G2019S LRRK2 CD63-pHluorin.tif]

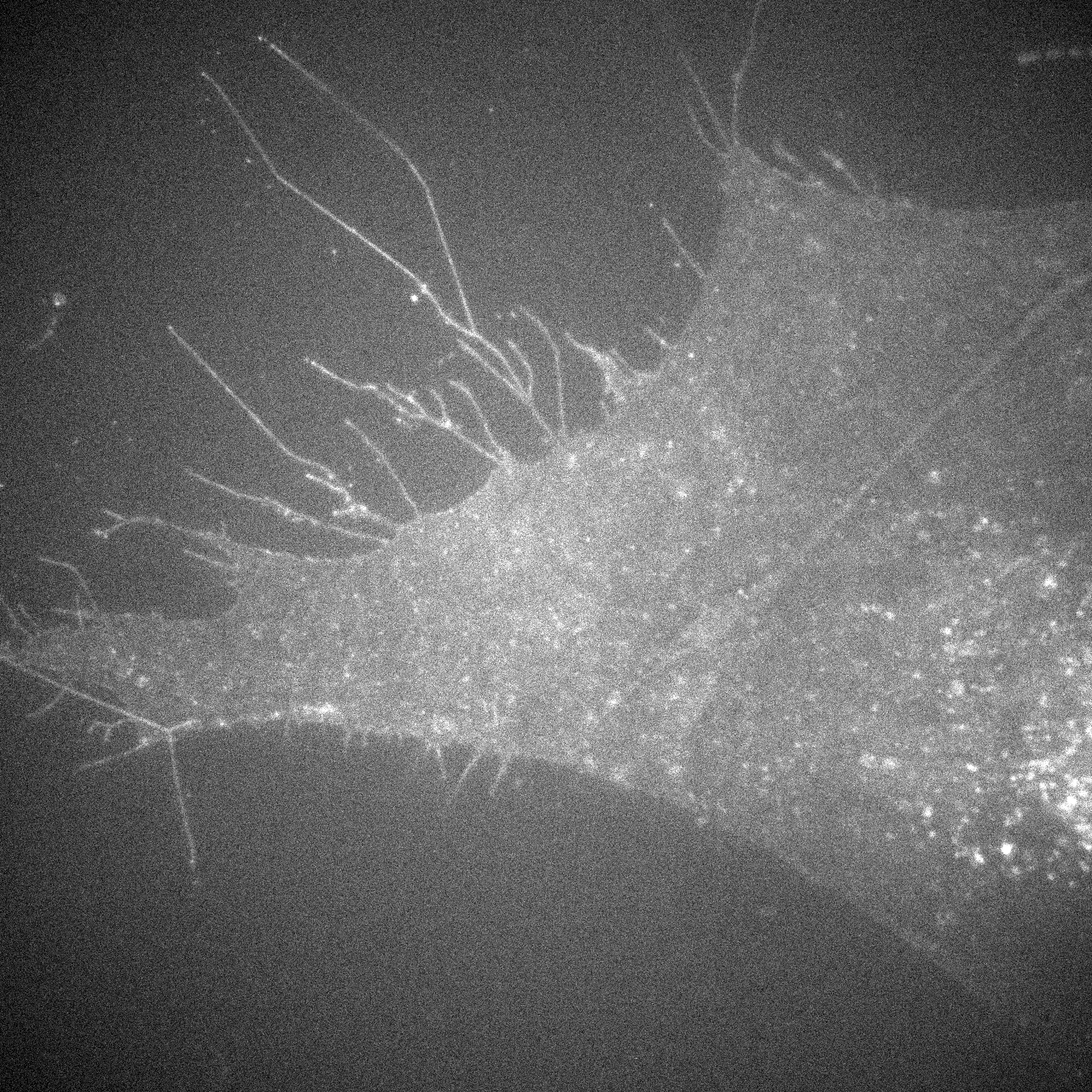

Supplement: Figure 7—source data 4. [file elife-106330-fig7-data4.zip › Figure 7H - Source data 1_EPI G2019S LRRK2 CD63-pHluorin.tif]

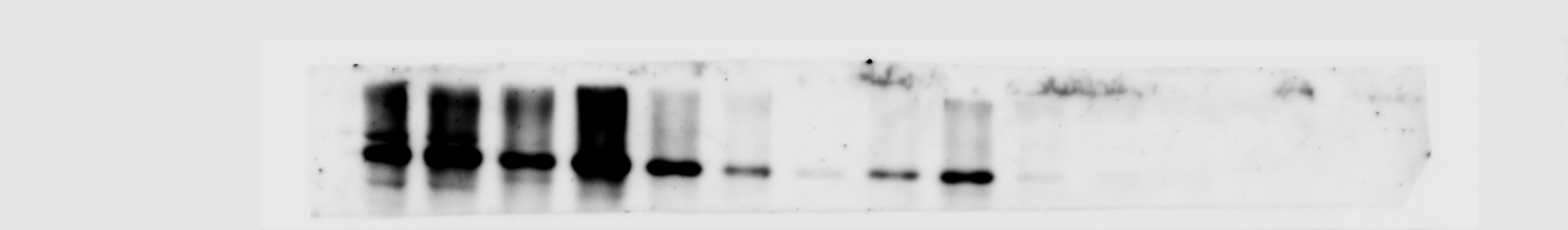

Supplement: Figure 7—figure supplement 1—source data 1. [file elife-106330-fig7-figsupp1-data1.zip › Figure 7A-figure supplement 1 - Source data 1.tif]

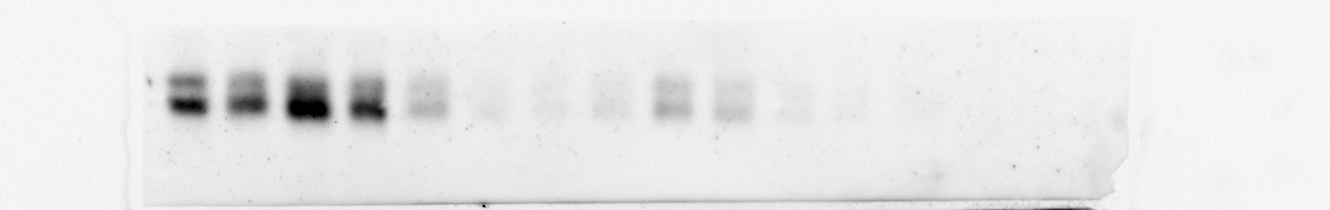

Supplement: Figure 7—figure supplement 1—source data 1. [file elife-106330-fig7-figsupp1-data1.zip › Figure 7A-figure supplement 1 - Source data 2.tif]

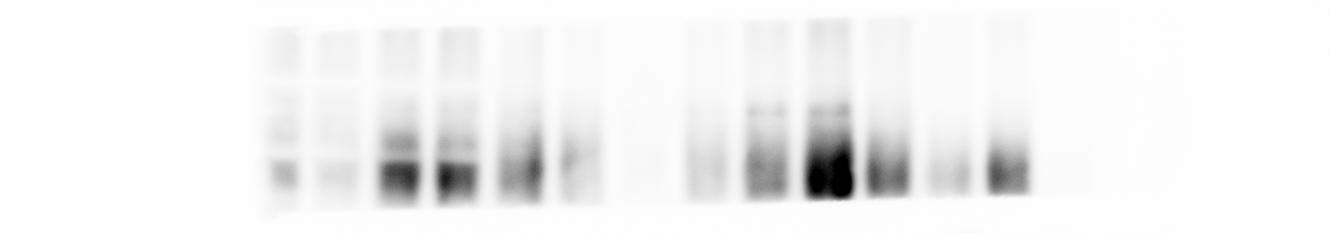

Supplement: Figure 7—figure supplement 1—source data 1. [file elife-106330-fig7-figsupp1-data1.zip › Figure 7A-figure supplement 1 - Source data 3.tif]

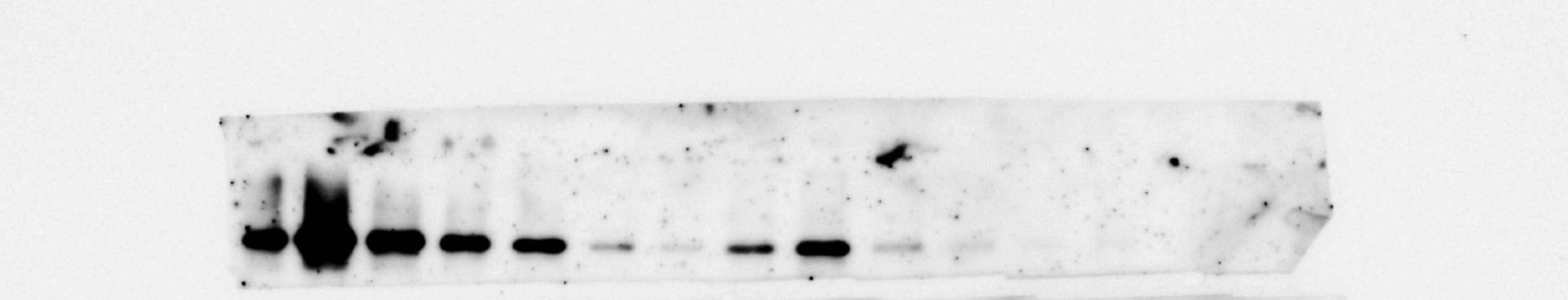

Supplement: Figure 7—figure supplement 1—source data 1. [file elife-106330-fig7-figsupp1-data1.zip › Figure 7A-figure supplement 1 - Source data 5.tif]

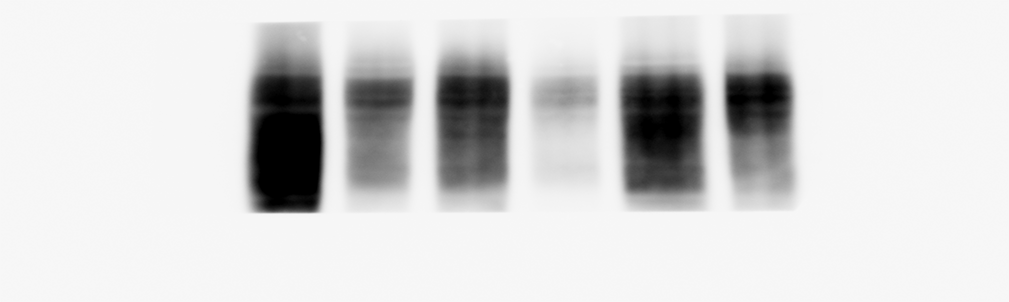

Supplement: Figure 7—figure supplement 1—source data 1. [file elife-106330-fig7-figsupp1-data1.zip › Figure 7B-figure supplement 2 - Source data 1.tif]

Figure 7A-figure supplement 1

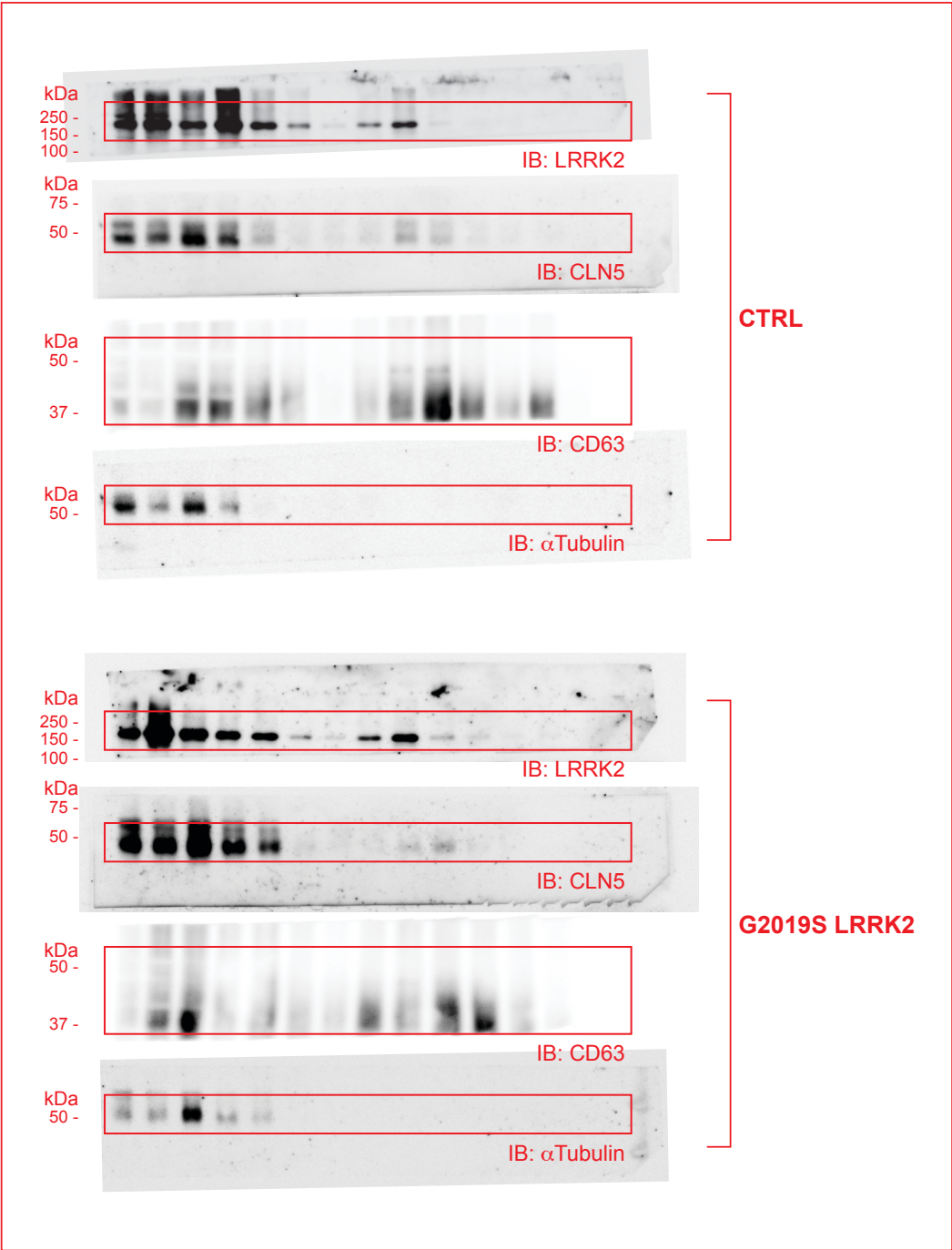

Figure 7B-figure supplement 1

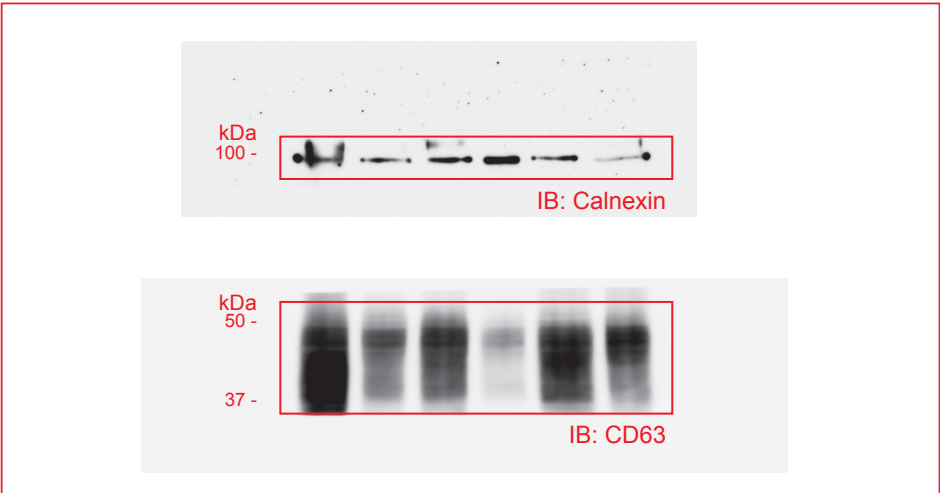

Supplement: Figure 7—figure supplement 1—source data 2. [file elife-106330-fig7-figsupp1-data2.zip › Figure 7-figure supplement 1 - Annotated Uncropped Blots.pdf]
